# High production volume chemicals in pre-cooked seafood-based meals consumed by inhabitants of Tarragona (Catalonia, Spain): presence and risk assessment

**DOI:** 10.1007/s00216-026-06521-2

**Published:** 2026-04-25

**Authors:** Laura Borrell, Francesc Borrull, Carme Aguilar, Eva Pocurull

**Affiliations:** Department of Analytical Chemistry and Organic Chemistry, Universitat Rovira I Virgili, Sescelades Campus, Marcel·Lí Domingo 1, 43007 Tarragona, Spain

**Keywords:** Pre-cooked seafood-based meals, High production volume chemicals, QuEChERS, GC-MS/MS, Dietary exposure, Risk assessment

## Abstract

High production volume chemicals (HPVCs) are widely used in industrial and consumer products and can migrate into food during processing and packaging. In this study, five families of HPVCs—phthalate esters (PAEs), organophosphate esters (OPEs), benzothiazoles (BTHs), synthetic musks (MUSKs), and benzenesulfonamides (BSAs)—were determined for the first time in 18 commercially available pre-cooked seafood meals representative of common consumption in Catalonia (Spain). All target compounds were detected in at least one sample, with an overall concentration trend of ΣPAEs > ΣOPEs > ΣBTHs > ΣMUSKs > ΣBSAs. PAEs were the dominant contaminants, largely driven by high concentrations of di-n-octylphthalate in two hake-based dishes. In general, contaminant levels in pre-cooked meals were higher than those previously reported for raw seafood from the same region, suggesting that processing and packaging may contribute to contamination through compound migration. OPEs, MUSKs, and BSAs showed higher concentrations in high-lipid species, whereas PAEs were more abundant in low-lipid species. Dietary exposure was estimated for six population groups under three exposure scenarios. Senior men showed the highest exposure, mainly due to higher seafood consumption. Despite the presence of these contaminants, risk assessment indicated that both non-carcinogenic and carcinogenic risks remain well below levels of concern. Overall, the results suggest that the analyzed HPVCs in pre-cooked seafood meals do not represent a significant health risk under the studied conditions, although packaging and processing may influence contaminant levels.

**Supplementary Information:**

The online version contains supplementary material available at https://doi.org/10.1007/s00216-026-06521-2.

## Introduction

Seafood consumption is widely recognized for its health-promoting properties, as it is rich in essential nutrients and represents an excellent source of high-quality proteins, polyunsaturated fatty acids, and vitamins. In particular, omega-3 fatty acids have been associated with a reduced risk of cardiovascular disease; in this regard, several studies have reported correlations between fish consumption and a lower incidence of coronary heart disease [1–3]. However, despite these nutritional benefits, there is increasing concern regarding the presence of contaminants in seafood, as dietary intake represents an important exposure pathway, and these compounds may pose potential risks to human health [4–8].

While most studies have primarily focused on raw seafood, modern lifestyles, characterized by limited time availability, have led to a notable increase in the consumption of pre-cooked meals. In Spain, the consumption of pre-cooked meals increased by 1.5% between 2022 and 2023, with seafood-based meals accounting for 37% of total meal sales, making them the most consumed category. Seafood pre-cooked meals, and pre-cooked meals in general, are valued for their convenience and ease of use. These products are usually packaged in multilayer materials, often containing plastics or plastic-coated components. Such materials may release contaminants into food through migration during cooking, storage, or reheating. Therefore, assessing contaminants in pre-cooked meals requires considering not only the contaminants originally present in raw food, but also the combined influence of cooking processes and packaging to achieve in this way a more realistic evaluation of the exposure and risk for consumers. ‬‬‬‬‬‬‬‬‬‬‬‬‬‬‬‬

Regarding sources of contamination, the current demand for goods such as electronic devices, furniture, plastics, personal care products, and household items to meet societal needs results in the large-scale production of pollutants. Among these substances, high production volume chemicals (HPVCs)—defined by the United States Environmental Protection Agency (EPA) as chemicals produced in amounts exceeding 500 tons per year [6]—are of particular concern. Due to their high production volumes and physicochemical properties, these compounds can readily enter the environment through emissions during manufacturing, product use, and waste management. As a result, many have been frequently detected in various environmental matrices, including indoor air, biota, sediments, wastewater, and downstream water bodies [6, 10, 11]. Once in the aquatic environment, they can bioaccumulate in seafood and subsequently enter the human body through dietary exposure [12].

HPVCs comprise a wide range of chemical families; however, the present study focuses on those considered most relevant. Phthalate esters (PAEs), widely used as plasticizers in industries producing electronics, toys, textiles, printing materials, inks, paints, and food-contact materials such as packaging and films, have been associated with endocrine disruption effects, asthma exacerbation, and adverse effects on the male reproductive system [12, 13]. Organophosphate esters (OPEs), used in numerous applications as flame retardants and plastic modifiers, have been linked to potential health effects, including kidney damage, reproductive issues, carcinogenic risks, and endocrine-disrupting effects [14, 15].

Benzenesulfonamides (BSAs) frequently used in the synthesis of disinfectants and dyes, and also used as plasticizers and fungicides in paints and coatings, can reach aquatic environments mainly through industrial runoff and may not be efficiently removed in pass through wastewater treatment plants (WWTPs) [16]. Benzothiazoles (BTHs), used in various applications, including paper production, textiles, plastics, and pesticides [17] have been associated with potential carcinogenic risk and endocrine-disrupting activity [18, 19]. Synthetic musks (MUSKs), commonly used as fragrances in personal care and cleaning products [20], have been detected in environmental compartments, as well as in the blood and tissues of mammals and aquatic organisms, and are of toxicological concern [21]. Among the compounds described, PAEs, OPEs, and MUSKs are relatively volatile, facilitating their dispersion in air, whereas BSAs and BTHs are more lipophilic, promoting their accumulation in lipid-rich matrices and their presence in surface waters and sediments [11, 22].

These findings highlight the need to monitor the presence of HPVCs in seafood. However, as noted previously, most studies conducted to date have focused on raw seafood [16, 22–26]. For example, Picot Groz et al. [20] and Saraiva et al. [21] determined synthetic fragrances in raw marine mussels from the Portuguese coast, and in raw oysters, mussels, salmon, and glass eels from southern Europe, respectively. Both studies employed QuEChERS (Quick, Easy, Cheap, Effective, Rugged, and Safe) extraction coupled with gas chromatography-mass spectrometry (GC-MS). Musk ketone was detected in almost all samples, while galaxolide and tonalide were quantified at concentrations up to 96.4 ng g^−1^ dry weight (dw) and 6.85 ng g^−1^ dw, respectively.

Other studies have focused on the determination of benzothiazoles and benzenesulfonamides in raw cod, salmon, mackerel, mussel, hake, sardine, tuna, shrimp, and squid [16, 17, 22], using extraction techniques such as QuEChERS, solid-phase microextraction, or ultrasound-assisted extraction, followed by gas or liquid chromatography coupled to mass spectrometry. The maximum concentrations reached were 43 ng g^−1^ wet weight (ww) for BTHs and 82 ng g^−1^ dw for BSAs. BSAs in squid samples were at the same order of magnitude as musk fragrances, but lower concentrations were generally observed for BSAs in most species analyzed. In contrast, OPEs and PAEs are typically present over a broader concentration range, and often at higher levels than other HPVCs in raw seafood. These compounds are commonly extracted using the techniques mentioned above, with their determination mainly performed by GC-MS [6, 14, 26, 27]. Reported OPE concentrations include values up to 130.8 ng g^−1^ ww in tuna, 623.6 ng g^−1^ lipid weight (lw) in mussels, and 1878.3 ng g^−1^ dw in sea bass [14, 26–28]. Similarly, PAEs have been reported at concentrations up to 221.73 ng g^−1^ ww in sardine and 1696.6 ng g^−1^ dw in European squid in previous studies [28] conducted by our research group.

In contrast, studies focusing on cooked seafood remain scarce, and most have examined the effect of cooking processes under controlled laboratory conditions on HPVC concentrations. For instance, Fierens et al. [23] investigated the effect of frying and grilling on phthalate concentrations in salmon. While frying did not substantially alter PAE concentrations, grilling led to a slight decrease for most PAEs, except for DEHP (di(2-ethyl-hexyl) phthalate), whose concentration increased 28-fold after grilling. Other studies have demonstrated that packaging can also be a source of contamination, showing that OPEs may migrate from packaging materials into seafood through direct contact or via oils present in the food matrix [8, 29].

Overall, available evidence indicates that multiple factors—including seafood origin, cooking processes, and packaging—may influence HPVC concentrations in seafood-based meals. Nevertheless, data remains limited, and further investigation is required. The present study aims to expand current knowledge on the occurrence of 29 HPVCs belonging to the BTHs, BSAs, OPEs, PAEs, and MUSKs families in pre-cooked seafood-based meals, to improve the assessment of the potential human health risks associated with their consumption. To this end, 18 frozen and fresh meals containing the seafood species most commonly consumed by the Spanish population were analyzed using a previously developed method based on QuEChERS extraction, followed by LipiFiltr clean-up and final determination by GC-MS/MS [26]. To the best of our knowledge, this is the first study evaluating the occurrence of this broad range of HPVCs in commercially available pre-cooked seafood-based meals.

## Materials and methods

### Reagents and materials

The present study determines the presence of 29 HPVCs, including 5 benzothiazoles (BTHs), 5 benzenesulfonamides (BSAs), 9 organophosphate esters (OPEs), 6 phthalate esters (PAEs), and 4 synthetic fragrances (MUSKs). The selected BTHs were 1-H-benzothiazole (BTH), 2-chlorobenzothiazole (ClBT), 2-(methylthio)-benzothiazole (MeSBT), 2-amino-H-benzothiazole (NH_2_BT), and 2-hydroxybenzothiazole (OHBT). The BSAs included benzenesulfonamide (BSA), ortho-toluenesulfonamide (o-TSA), N-methyl-para-toluenesulfonamide (Me-p-TSA), N-ethyl-para-toluenesulfonamide (Et-p-TSA), and para-toluenesulfonamide (p-TSA). The OPEs comprised triethyl phosphate (TEP), tributyl phosphate (TBP), tri-isobutyl phosphate (TiBP), tris(2-chloroisopropyl) phosphate (TCPP), tris(2-chloroethyl) phosphate (TCEP), tris(2-ethylhexyl) phosphate (TEHP), 2-ethylhexyl-diphenyl phosphate (EHDPP), triphenyl phosphate (TPP), and tritolyl phosphate (TTP). The PAEs considered were dimethylphthalate (DMP), diethylphthalate (DEP), di-iso-butylphthalate (DiBP), diethylhexyladipate (DEHA), di-(2-ethylhexyl)phthalate (DEHP), and di-n-octylphthalate (DnOP). The musks included cashmeran (DMPI), traseolide (ATII), galaxolide (HHCB), and tonalide (AHTN).

A deuterated internal standard (IS) was used for each chemical family: d_4_-benzothiazole (d_4_-BTH), 4-tolyl-d_4_-sulfonamide (d_4_-p-TSA), d_27_-tributylphosphate (d_27_-TBP), d_4_-diethylhexyl-phthalate (d_4_-DEHP), and d_15_-musk xylene (d_15_-MX). All analytical standards listed above had a purity higher than 98% and were purchased from Sigma-Aldrich (St. Louis, MO, USA), except for d_4_-p-TSA, d_4_-BTH, d_15_-MX, and ATII, which were obtained from LGC Standards (Barcelona, Spain).

Methanol (RS-PESTIPUR grade) and ethyl acetate (RS-PESTIPUR grade) were purchased from Carlo Erba (Barcelona, Spain). Acetonitrile (ACN) and acetone (HPLC gradient grade) were acquired from Fisher Scientific (Waltham, MA, USA). Ultrapure water was produced using a Synergy water purification system (Millipore, MA, USA). Original QuEChERS salt extraction kits (Original Sachets) and 0.22-mm PTFE syringe filters (13 mm) were obtained from Scharlab S.L. (Barcelona, Spain). LipiFiltr cartridges were supplied by Carlo Erba (Barcelona, Spain). Helium and nitrogen gases with purities higher than 99.999% were provided by Carburos Metálicos (Tarragona, Spain).

### Sample collection

A total of 18 pre-cooked seafood meals were purchased from local markets and grocery stores in Tarragona (Catalonia, Spain). The selected meals included seven species among the ten most consumed seafood species in Catalonia, according to the ENCAT 2003 survey [30]: squid (*Loligo vulgaris*), salmon (*Salmo salar*), mussel (*Mytilus edulis*), shrimp (*Parapenaeus longirostris*), sardine (*Sardina pilchardus*), hake (*Merluccius merluccius*), and cod (*Gadus morhua*). Two different dishes were selected per species, except for hake and cod, for which three and five dishes were purchased, respectively, to cover the range of cooking methods.

Species were also classified according to lipid content to include both low-lipid species (cod, hake, squid, mussels, and shrimp, containing 0.5 g, 1 g, 1.3 g, 1.9 g, and 1.4 g of fat per 100 g, respectively) and high-lipid species (salmon and sardine, containing 12 g and 7.5 g of fat per 100 g, respectively) [31].

Samples were grouped into three pre-processing categories: freshly cooked (prepared daily and sold in bulk), refrigerated (sold pre-packaged in individual portions), and frozen. Each meal was packaged in plastic-based containers, with different packaging formats represented across samples. A detailed description of each meal is provided in Table 1.

**Table 1:** Seafood species, product description, type of pre-process and packaging type used for each one of the pre-cooked seafood meals

Seafood species	Sample	Product description	Type pre-process	Packaging
**Squid**	**S1**	Stuffed squid in sauce	Freshly cooked	Plastic takeaway container
	**S2**	Grilled baby squid with confit potatoes	Refrigerated	Plastic-coated cardboard tray
**Shrimp**	**S3**	Boiled shrimp	Refrigerated	Plastic container
	**S4**	Shrimp “al ajillo”	Frozen	Plastic casserole
**Salmon**	**S5**	Steamed salmon with couscous and vegetables	Refrigerated	Plastic container
	**S6**	Smoked salmon	Refrigerated	Plastic, vacuum-sealed
**Sardine**	**S7**	Smoked sardines in sunflower oil	Refrigerated	Plastic container
	**S8**	Fried sardines in pickling sauce	Freshly cooked	Plastic takeaway container
**Mussel**	**S9**	Mussels cooked in Albariño sauce	Refrigerated	Plastic casserole
	**S10**	Mussels in their own juice	Frozen	Plastic, vacuum-sealed
**Hake**	**S11**	Fried hake in onion and shrimp sauce	Freshly cooked	Plastic takeaway container
	**S12**	Steamed hake with rice and shrimps in sauce	Frozen	Plastic and aluminum container
	**S13**	Fried and breaded hake	Frozen	Plastic bag
**Cod**	**S14**	Steamed cod with quinoa and vegetables	Refrigerated	Plastic container
	**S15**	Fried cod in onion sauce	Freshly cooked	Plastic takeaway container
	**S16**	Oven-baked cod with potatoes, asparagus and onion	Freshly cooked	Plastic takeaway container
	**S17**	Fried cod with “samfaina”	Refrigerated	Plastic and aluminum container
	**S18**	Shredded salt raw cod salad, “esqueixada”	Freshly cooked	Plastic takeaway container

### Sample pretreatment

Pre-cooked meals were removed from their packaging, and the seafood portions were mechanically separated from sauces and condiments as thoroughly as possible. The seafood samples were subsequently freeze-dried using a miVac Duo freeze-drying system from Genevac (Ipswich, UK) and homogenized by grinding. Each sample was weighed before and after lyophilization to determine moisture content. The resulting dried homogenates were stored in glass containers at −24 °C until further analysis.

### Analytical method

The analytical methodology was based on the procedure described by Castro et al. [26]. Briefly, 0.1 g of lyophilized sample was placed in a glass centrifuge tube and mixed with 10 mL of ACN and 10 mL of ultrapure water. The mixture was vortexed to ensure homogenization. QuEChERS extraction was then performed by adding the QuEChERS Original salt mixture to the tube, followed by vortex agitation for 5 min and subsequent centrifugation. The organic phase was filtered using a LipiFiltr device and evaporated to near dryness. Extracts were reconstituted to a final volume of 2 mL with ethyl acetate, and the IS mixture was added to achieve a final concentration of 50 ng mL^−1^.

Instrumental analysis was carried out using an Agilent 8890 GC system coupled to an Agilent 7000D triple quadrupole mass spectrometer (Agilent Technologies, Palo Alto, CA, USA), equipped with a PAL RSI 120 autosampler (CTC Analytics, Zwingen, Switzerland). A 25 μL aliquot of the QuEChERS extract was injected using an Agilent Multi-Mode Inlet (MMI) operated in solvent vent mode. Helium was used as the carrier gas at a constant flow rate of 1.2 mL min^−1^. The injector temperature program started at 75 °C (held for 0.5 min) and was increased to 325 °C at 600 °C min^−1^, and was held until the end of analysis.

Chromatographic separation was achieved using a ZB-50 capillary column (30 m × 25 mm internal diameter, 0.25 μm film thickness) from Phenomenex. The oven temperature program was as follows: initial temperature of 75 °C (held for 2.87 min), ramped to 300 °C at 15 °C min^−1^, with a final hold of 10 min, resulting in a total run time of 27.87 min. The transfer line was set at 280 °C.

The MS system operated in electron ionization mode at 70 eV. The ion source temperature was set at 230 °C, and both quadrupoles were maintained at 150 °C. Data acquisition and processing were performed using Agilent MassHunter Workstation software (Quantitative and Qualitative Analysis, version 10.0). Retention times, quantitative and qualitative transitions, and collision energies for each target analyte are provided in Supplementary Material (Table S1).

### Quality assurance and quality control (QA/QC)

Given the potential presence of target compounds in both the environment and laboratory materials, strict contamination control measures were performed, following procedures described in previous studies [6, 26]. Plastic materials were avoided whenever possible throughout the extraction process; for example, glass Falcon tubes were used for QuEChERS extraction. All glassware was cleaned by immersion in isopropanol and sonication for 30 min. Periodic method blanks and instrumental blanks were included, and background signals were subtracted when necessary. Additionally, quality control standards were analyzed every five injections. The chromatographic injection needle was rinsed three times with methanol and ethyl acetate before and after each injection.

### Exposure and risk assessment

Human exposure values (*E*_t_) were calculated to estimate dietary exposure to contaminants from each pre-cooked meal. *E*_t_ was calculated using Eq. 1 [32], where *C*_f_ is the mean daily consumption of seafood species *f*, and *X*_t,f_ is the concentration of compound *t* in species *f* (ng g^−1^ ww). Daily consumption values (*C*_f_, g day^−1^ ww) were obtained from the most recent ENCAT survey [30], which provides seafood consumption data in Catalonia, segregated by seafood species, human gender, and age (values are provided in Supplementary Material, Table S2). Consumption values, *C*_f_, were normalized by the human body weight (bw) in kg before use.

1 $${E}_{\mathrm{t} }=\sum\nolimits_{f =1}^{p}{C}_{\mathrm{f}}{X}_{\mathrm{t},\mathrm{f}}$$

Risk assessment for non-genotoxic, non-carcinogenic compounds was performed using the acceptable daily intake (ADI) approach, which is based on the non-observed-adverse-effect-level (NOAEL). The NOAEL represents the highest exposure level at which no adverse effects are observed in the test species. ADI values are calculated by dividing the NOAEL by an uncertainty factor of 100, which counts for potential differences between humans and animals in long-term exposure studies. The risk factor (*R*_t_) was subsequently calculated by comparing the human exposure values to the ADI, as shown in Eq. 2. A value of *R*_t_ > 100 indicates that the estimated exposure exceeds the ADI.

2 $${R}_{\mathrm{t}}=\frac{{E}_{\mathrm{t}}}{{\mathrm{ADI}}_{\mathrm{t}}}\times 100$$

For carcinogenic compounds, the margin of exposure (MOEt) was calculated using Eq. 3, based on the benchmark dose (BMD) approach. The BMD represents the lower confidence limit of the dose at which adverse effects can be observed in approximately 5–10% of experimental animals, which is then compared to human exposure values. According to the European Food Safety Authority (EFSA) [33], a risk is considered low when MOEt > 10,000, whereas values below 10,000 suggest that the potential risk may exist and further evaluation is required.

3 $${\mathrm{MOE}}_{\mathrm{t}}=\frac{{\mathrm{BMD}}_{\mathrm{t}}}{{E}_{\mathrm{t}}}$$

To handle compounds that were not detected or were below the limit of quantification (LOQ), EFSA recommends defining three scenarios for human exposure calculations: lower-bound (LB), middle-bound (MB), and upper-bound (UB). In the LB scenario, non-detected compounds are assumed to be zero, and below-LOQ compounds are set to the limit of detection (LOD). In the MB scenario, non-detected compounds are set to half the LOD, and below-LOQ compounds to half the LOQ. In the UB scenario, non-detected compounds are set to the LOD, and below-LOQ compounds to the LOQ.

## Results and discussion

### Method quality parameters

The analytical method applied in this study was based on a procedure previously developed by our research group [6]. As the original method was designed for the analysis of raw seafood matrices, it was validated to assess its suitability for the pre-cooked seafood matrices analyzed in the present study.

Instrumental quality parameters were first evaluated in terms of linearity, limits of detection (LODs), and limits of quantification (LOQs) through the analysis of standard solutions. All compounds exhibited satisfactory linearity (*r*^2^ > 0.9932) within the studied concentration ranges, which for most analytes extended from 0.005 to 250 µg L^−1^. LODs were defined as the concentrations providing a signal-to-noise ratio greater than 3, while the lowest point of the linear ranges was taken as the LOQ. These values are summarized in Table S3.

Method quality parameters were subsequently evaluated by determining apparent recoveries (Rapp), matrix effects (ME), method detection limits (MDLs), and method quantification limits (MQLs) for each compound and meal type, due to the variability in sample composition. Apparent recoveries were obtained by spiking pretreated samples (*n* = 3, RSD < 20%) at 1 mg kg^−1^ dw prior to extraction. Rapp values ranged from 28% to approximately 100%. Considerable variability was observed for individual analytes depending on the type of meal. For instance, galaxolide showed an apparent recovery of 28% in S14 (steamed cod with quinoa and vegetables), whereas values as high as 96% were observed in S18 (shredded salt raw cod salad, “esqueixada”). These findings indicate that, in addition to the cooking process, the presence of condiments and additional ingredients influences extraction efficiency and matrix effects, thereby affecting apparent recoveries. Nevertheless, despite the complexity of the matrices, apparent recoveries for most analytes were not lower than 35%, which is consistent with values commonly reported for raw seafood matrices in previous studies [34].

Matrix effects were evaluated by spiking pretreated samples (*n* = 3, RSD < 20%) at 1 mg kg^−1^ dw after the extraction. In agreement with the results obtained for Rapp, ME values varied considerably among individual analytes and meal types, leading to both signal suppression and signal enhancement. This behavior is consistent with the compositional variability of pre-cooked seafood meals. MDLs and MQLs were estimated based on the instrumental quality parameters, apparent recoveries, and the extraction procedure. For most analytes, MDLs and MQLs were in the low ng g^−1^ ww range, as detailed in Table S3. These results are consistent with those reported in previous studies analyzing raw and cooked seafood [12, 16, 35].

### General trends in HPVC concentrations

All target HPVCs were detected above the MQLs in at least one of the analyzed samples, except for two BTHs (OHBT and NH_2_BT), which were either not detected or present at concentrations below the MQLs (Table 2). Detection frequencies varied considerably among compounds, ranging from 6% (OHBT) to 89% (TiBP). MeSBT, TBP, TiBP, and HHCB were the most frequently detected analytes (DF > 83%), consistent with their widespread use and, in some cases, their relatively high persistence and slow degradation rates, particularly for certain OPEs [5, 36].

**Table 2:** Concentration (ng g^−1^ ww) of HPVCs in the different seafood-based dishes (S1–S18), detection frequency (DF %), and geometric mean (GM) along with sum of concentrations by chemical family in each individual dish, and the range

Compound	S1	S2	S3	S4	S5	S6	S7	S8	S9	S10	S11	S12	S13	S14	S15	S16	S17	S18	DF	GM
BTH	76.4	*	*	*	n.d.	< MQL	33.2	42.7	*	< MQL	n.d.	75.2	n.d.	n.d.	*	*	n.d.	n.d.	33	53.4
ClBT	*	*	*	*	1.75	*	5.61	4.47	*	1.68	< MQL	< MQL	*	n.d.	n.d.	*	n.d.	n.d.	33	2.93
MeSBT	19.7	45.4	*	22.4	23.2	12.5	32.4	14.8	9.01	10.5	*	92.4	7.56	28.1	n.d.	316	28.8	4.99	83	22.8
OHBT	n.d.	*	n.d.	< MQL	n.d.	*	*	*	*	*	n.d.	n.d.	n.d.	n.d.	n.d.	n.d.	n.d.	n.d.	6	n.d.
NH_2_BT	< MQL	< MQL	n.d.	n.d.	n.d.	n.d.	n.d.	n.d.	n.d.	n.d.	< MQL	*	n.d.	n.d.	n.d.	n.d.	n.d.	*	17	n.d.
BSA	< MQL	n.d.	9.66	n.d.	< MQL	*	n.d.	*	*	*	n.d.	n.d.	n.d.	n.d.	n.d.	n.d.	n.d.	n.d.	17	9.66
o-TSA	*	*	n.d.	21.5	*	*	*	*	*	*	*	n.d.	27.4	n.d.	n.d.	n.d.	< MQL	< MQL	22	24.3
Me-p-TSA	0.80	*	7.80	2.76	3.90	*	14.4	*	*	*	< MQL	< MQL	< MQL	n.d.	n.d.	*	n.d.	n.d.	44	3.96
Et-p-TSA	0.87	1.14	3.20	2.06	2.24	*	12.1	*	*	*	n.d.	< MQL	< MQL	n.d.	< MQL	< MQL	n.d.	< MQL	61	2.37
p-TSA	2.18	2.91	n.d.	3.20	4.15	*	n.d.	*	*	*	n.d.	n.d.	< MQL	n.d.	n.d.	n.d.	n.d.	n.d.	28	3.03
TEP	n.d.	0.69	*	3.90	*	*	*	*	19.6	12.2	< MQL	8.72	n.d.	n.d.	13.2	n.d.	n.d.	0.72	44	4.73
TBP	n.d.	2.44	4.09	1.46	2.08	7.70	13.1	7.67	6.72	3.70	< MQL	< MQL	n.d.	< MQL	n.d.	< MQL	< MQL	< MQL	83	4.37
TiBP	< MQL	< MQL	2.31	< MQL	0.92	2.99	6.39	5.26	5.37	2.37	< MQL	2.32	1.95	n.d.	< MQL	90.6	4.65	n.d.	89	4.08
TCPP	24.4	< MQL	26.5	< MQL	*	*	*	< MQL	26.8	20.8	n.d.	18.7	n.d.	n.d.	*	*	*	*	44	23.19
TCEP	n.d.	< MQL	< MQL	n.d.	1.48	3.92	< MQL	< MQL	1.27	0.69	n.d.	*	*	*	*	*	< MQL	n.d.	56	1.50
TEHP	2.07	< MQL	29.4	24.0	n.d.	72.4	5.18	55.7	2540	2123	n.d.	6.60	n.d.	< MQL	13.9	n.d.	n.d.	4.19	72	33.69
EHDPP	n.d.	*	4.97	2.13	*	*	*	*	5.19	2.67	n.d.	n.d.	< MQL	*	*	*	*	< MQL	33	3.48
TPP	n.d.	1.44	6.45	12.4	17.6	15.5	37.1	*	7.53	5.17	n.d.	*	*	*	*	*	*	*	56	9.06
TTP	45.3	12.8	9.20	3.47	5.67	10.4	27.1	*	23.1	13.2	n.d.	< MQL	11.6	< MQL	< MQL	*	*	n.d.	72	12.68
DMP	3.83	*	2.95	3.52	*	*	*	44.1	10.0	9.14	< MQL	n.d.	n.d.	n.d.	105	22.9	43.7	< MQL	61	13.69
DEP	35.0	*	72.4	37.1	43.0	34.9	121	40.2	61.6	48.2	8.86	n.d.	n.d.	n.d.	84.6	274	58.0	n.d.	72	52.70
DiBP	48.5	84.1	66.4	*	25.4	43.6	135	53.8	121	87.0	< MQL	*	n.d.	< MQL	51.7	*	105	51.1	78	65.6
DEHA	19.5	*	123	52.7	55.6	90.0	186	103	96.6	36.0	n.d.	n.d.	n.d.	< MQL	213	n.d.	*	n.d.	61	78.6
DEHP	< MQL	< MQL	*	*	*	247	*	*	*	91.6	n.d.	n.d.	n.d.	326	138	5.99	295	n.d.	44	110
DnOP	n.d.	n.d.	*	34.5	72.8	86.3	*	58.5	265	232	n.d.	3969	2668	*	*	n.d.	*	n.d.	44	232
DPMI	n.d.	n.d.	*	n.d.	n.d.	*	n.d.	n.d.	14.2	6.11	1.91	4.98	2.64	n.d.	*	28.4	37.7	*	39	8.12
ATII	n.d.	n.d.	n.d.	n.d.	1.69	n.d.	n.d.	4.85	n.d.	n.d.	< MQL	n.d.	< MQL	< MQL	< MQL	< MQL	< MQL	< MQL	50	2.86
HHCB	20.9	15.4	28.1	25.7	32.6	17.9	44.3	28.1	20.4	30.6	20.9	n.d.	n.d.	*	24.8	15.3	n.d.	15.0	83	23.12
AHTN	25.1	*	30.6	18.8	*	*	*	*	73.7	29.8	112	8.59	36.5	n.d.	< MQL	< MQL	58.5	34.3	67	34.26
Sum																			**Range**	
ΣBTHs	96.1	45.4	n.d.	22.4	24.9	12.5	71.3	62.0	9.01	12.2	< MQL	168	7.56	28.1	n.d.	316	28.8	4.99	n.d.–316	
ΣBSAs	3.85	4.05	20.7	29.5	10.3	*	26.6	*	*	*	< MQL	< MQL	27.4	n.d.	< MQL	< MQL	< MQL	< MQL	n.d.–29.5	
ΣOPEs	71.7	17.4	82.9	47.4	27.8	113	89.4	68.6	2635	2183	< MQL	36.3	13.5	< MQL	27.1	90.6	4.65	4.91	n.d.–2635	
ΣPAEs	106.8	84.1	265	128	197	502	441	230	555	504.0	8.86	3969	2668	326	592	303	502	51.1	n.d.–3969	
ΣMUSKs	46.0	15.4	58.7	44.4	34.3	17.9	44.3	33.0	108	66.5	135	13.6	39.1	< MQL	25	43.7	96.2	49.3	n.d.–135	

*n.d.*, not detected

< MQL concentration lower than method quantification limit

^*^Compound not determined due to very low Rapp

Individual concentrations for each meal (Table 2) indicate that most analytes were present at low ng g^−1^ ww levels. Notable exceptions were the organophosphate TEHP and the phthalate DnOP, which reached concentrations of up to 2540 ng g^−1^ ww and 3969 ng g^−1^ ww, respectively. The ranges of summed concentrations for each chemical family, together with the corresponding geometric mean (GM) values, were as follows: BTHs, n.d.–316 ng g^−1^ ww (GM: 18.5 ng g^−1^ ww, with BTH as the main contributor); BSAs, n.d.–29.5 ng g^−1^ ww (GM: 4.04 ng g^−1^ ww, with o-TSA as the predominant compound); OPEs, n.d.–2636 ng g^−1^ ww (GM: 8.02 ng g^−1^ ww, mainly TCPP and TEHP); PAEs, n.d.–3969 ng g^−1^ ww (GM: 64.4 ng g^−1^ ww; primarily DnOP, DEHP, and DEHA); and MUSKs, n.d.–135 ng g^−1^ ww (GM: 18.4 ng g^−1^ ww, mainly HHCB and AHTN). Among the chemical families, PAEs exhibited the highest geometric mean concentration, whereas BSAs showed the lowest, in agreement with trends previously reported for raw seafood species [6].

To the best of our knowledge, this is the first study addressing the occurrence of HPVCs in pre-cooked seafood-based meals. Therefore, comparisons with the literature are limited, as most previous studies have focused on raw seafood. In general, reported concentrations in raw seafood are similar to or lower than those obtained in the present study, except in cases where samples were collected from highly contaminated areas [6, 18, 22, 26, 37]. For instance, BTH concentrations up to 13,800 ng g^−1^ dw were reported in raw mollusks from the Bohai Sea (China), an area heavily impacted by industrial activities [18], whereas the maximum concentration observed in the present study was 92.4 ng g^−1^ ww for MeSBT. Conversely, BTH concentrations in raw seafood purchased from supermarkets have previously been reported at low ng g^−1^ ww levels [6], comparable to those observed in the present study.

Among the limited number of studies investigating cooked seafood, Fierens et al. [23] evaluated the effect of laboratory-controlled cooking processes on eight phthalates in salmon. In general, PAE concentrations decreased after cooking; however, contact with non-stick coatings during grilling resulted in increased PAE levels, reaching up to 4253 ng g^−1^ ww.

Other studies have focused on packaged seafood products, mainly stored in plastic containers or cans, targeting PAEs and OPEs. For instance, DEHP concentrations up to 5932 ng g^−1^ ww were reported in seafood packaged in plastic-based containers [12], whereas the highest DEHP concentration observed in the present study was 326 ng g^−1^ ww in a dish also packaged in a plastic container. Regarding OPEs, several studies have reported concentrations comparable to those observed here, particularly in canned fish, dried seafood (e.g., clams and shrimp), smoked herring fillets, and other processed products [8, 36]. Wang et al. [8] determined 12 OPEs in various fish products from multiple provinces in China, including canned, grilled, and processed fish items. The geometric mean of the summed OPE concentrations in 16 fish products was 5.34 ng g^−1^ ww, similar to the value obtained in the present study (GM: 8.02 ng g^−1^ ww). In contrast, lower overall mean OPE concentrations (up to 0.75 ng g^−1^ ww) have been reported in raw and processed crustaceans, fish, seafood, and mussels from Belgian markets [36].

Overall, the limited number of studies on cooked seafood, together with the variability in reported concentrations, suggests that multiple factors influence HPVC levels, including seafood origin, packaging materials, cooking methods, and preservation processes. Further investigation is therefore warranted.

**Fig. 1:**
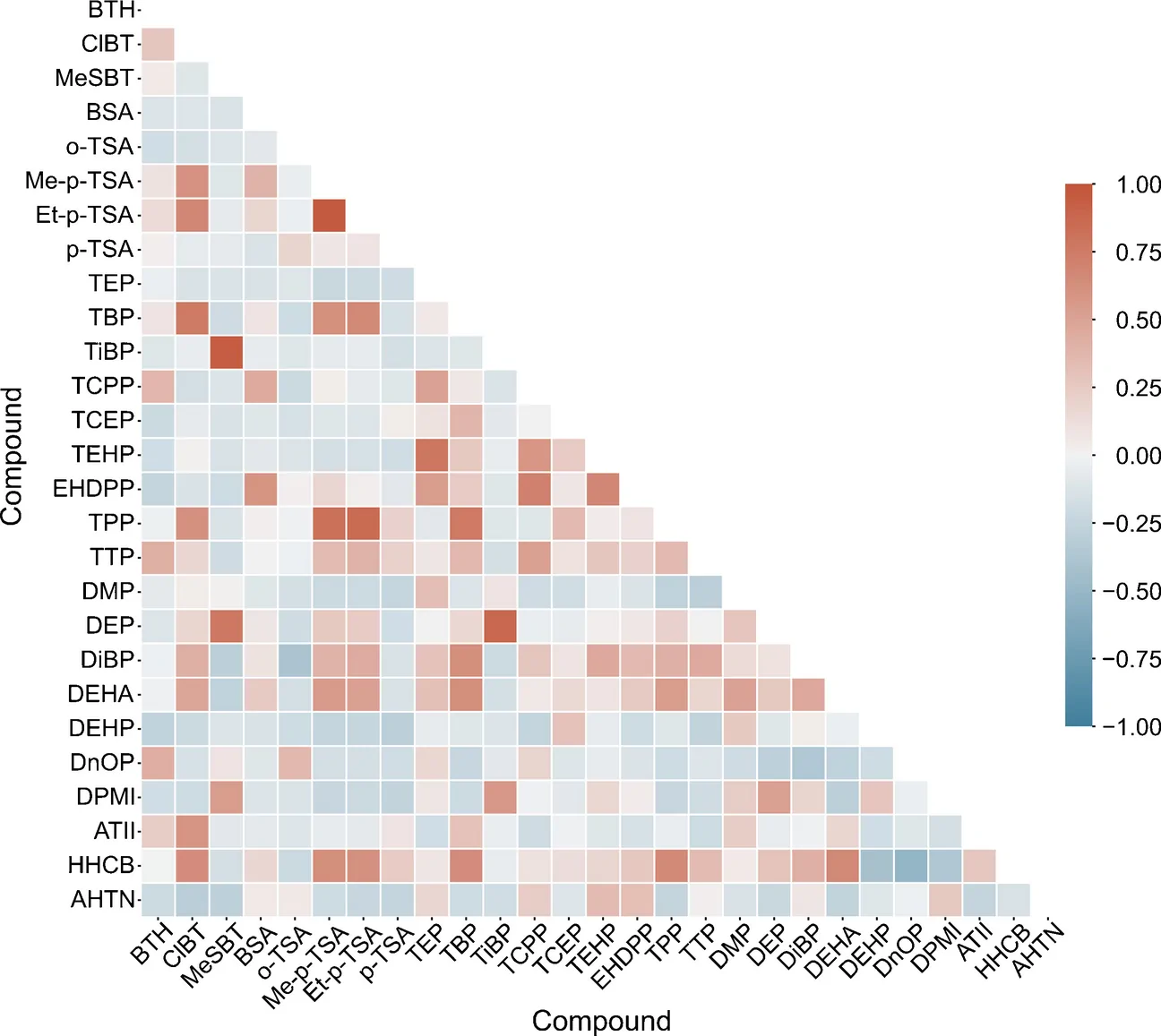
Pearson correlation matrix between the different HPVC concentrations

To explore potential correlations among HPVC concentrations in the present study, a Pearson correlation analysis was performed (Fig. 1).

Although most compounds exhibited weak or non-significant correlations, strong positive correlations were observed between Et-p-TSA and Me-p-TSA (both BSAs), between TiBP and DEP (an OPE and a PAE), and between MeSBT (a BTH) and the OPEs TEHP and TEP. Some of these compounds are used as co-plasticizers due to their structural similarities [35], and their correlation may reflect co-occurrence resulting from impurities originating from shared production processes. This observation suggests that mixtures of these compounds may be associated with materials used in the manufacturing of pre-packaged foods, particularly packaging materials [36]. Furthermore, the phthalate DEP and the organophosphate TPP showed strong correlations with several compounds belonging to different chemical families. Considering that both PAEs and OPEs are widely used in plastic packaging formulations [38], their presence and intercorrelations may indicate a common source related to packaging materials.

### Distribution patterns of HPVCs among the selected pre-cooked seafood types

The selection of pre-cooked seafood meals was based on the species most frequently consumed by the Catalan population, according to the ENCAT 2003 survey [30], and on their commercial availability. The selected dishes comprised seven species representing both low- and high-lipid content, and included a variety of cooking and preservation methods, namely frozen, refrigerated, and freshly cooked products. Although packaging formats differed among products, most were packaged in plastic-based materials.

**Fig. 2:**
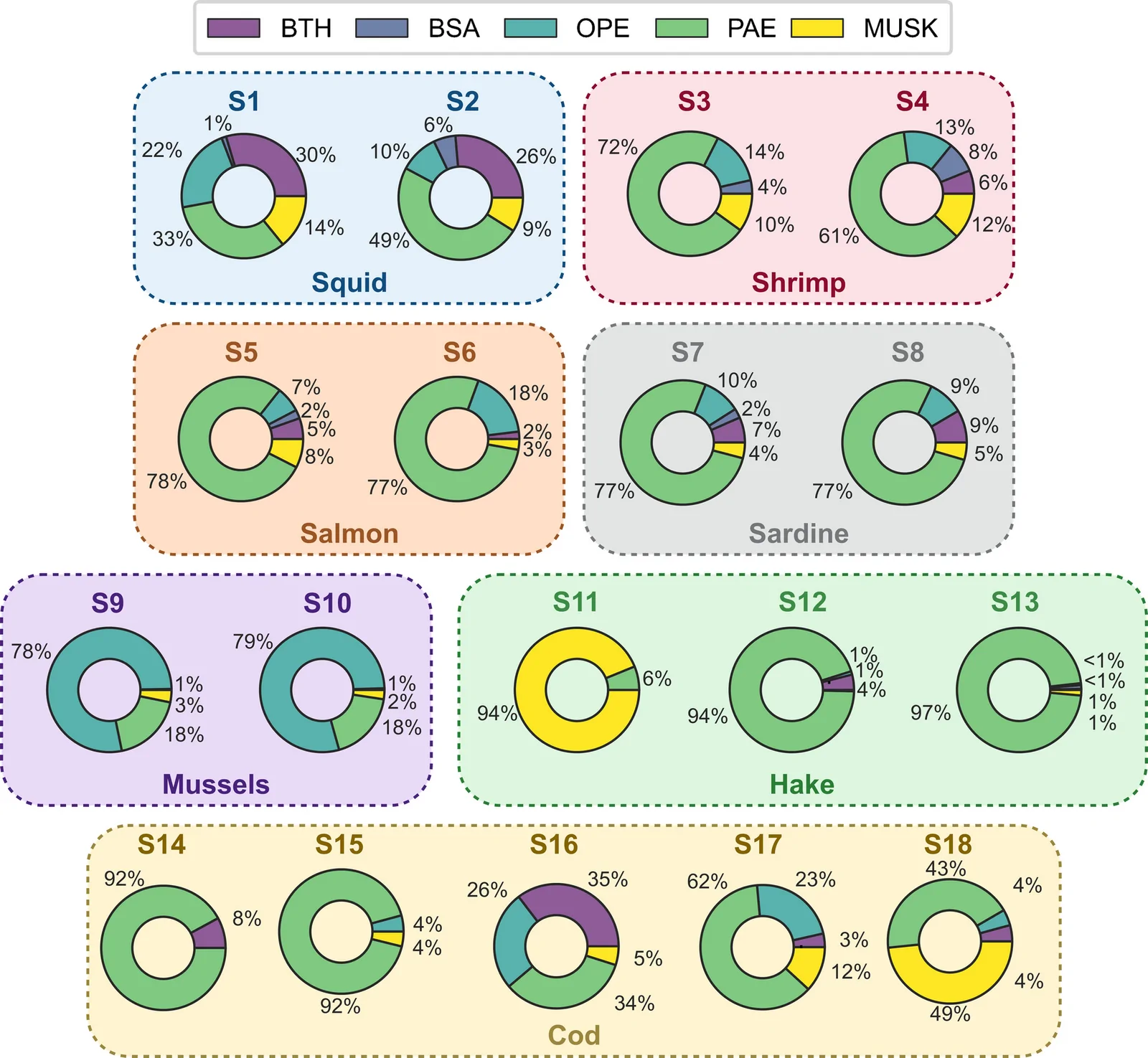
Total contribution of each HPVC family in each pre-cooked seafood meal

Figure 2 presents each meal as a pie chart illustrating the relative contribution of each chemical family to the total HPVC concentration. As a general trend, squid-, shrimp-, salmon-, and sardine-based meals showed a predominance of PAEs, contributing up to 78% of the total concentration in S5 (salmon-based). Mussel-based meals exhibited the highest contribution of OPEs (up to 79% in S10), whereas hake- and cod-based meals displayed greater composition variability; nevertheless, PAEs generally remained the dominant chemical family.

Squid-based meals (S1 and S2) showed similar contributions across chemical families, regardless of differences in cooking method or packaging material. In both cases, PAEs were the predominant family, although OPEs contributed more substantially in S1 than in S2. Shrimp-based meals (S3 and S4) also presented comparable distribution patterns, following the order PAEs > OPEs > BSAs > MUSKs, while BTHs were generally not quantified due to low Rapp. Salmon- and sardine-based meals (S5–S8) were likewise dominated by PAEs, with only minor variations in the contribution of other families. BSAs were below the LOQ in S6 (salmon) and S8 (sardine). In contrast, mussel-based meals (S9 and S10) displayed a distinct distribution characterized by OPEs predominance (OPEs > > PAEs > MUSKs > BTHs). This pattern was largely driven by high TEHP concentration, 2540 and 2123 ng g^−1^ ww, in S9 and S10, respectively.

No consistent contribution patterns were observed among hake- and cod-based meals. For hake, samples S12 and S13 exhibited a clear contribution of PAEs (> 90% of total HPVCs), whereas MUSKs were predominant in S11 (94%). Notably, S12 and S13 were frozen products, while S11 was freshly cooked, which may have facilitated greater environmental exposure to MUSKs, as also observed in S18 (freshly cooked cod-based meal). Cod-based meals displayed the greatest variability among all the species. Although PAEs were generally the major contributors, the relative contribution of each family varied markedly between samples.

Regarding absolute concentrations (Figure S4, Supplementary Material), mussel-based (S9 and S10) and hake-based (S12 and S13) meals exhibited substantially higher total HPVC levels than the other samples. In mussels, this increase was mainly due to TEHP, whereas in hake-based meals, it was largely attributable to DnOP.

Overall, PAEs were the most contributing family in most meals, except for mussel-based dishes (S9 and S10), and certain cod-based meals (S16, S18). All chemical families were detected in most samples, except for BSAs and BTHs, which were frequently below the LOQ. Although species-related patterns were observed, no consistent trends were identified according to cooking method or packaging materials which could explain the observed variability. Nevertheless, the consistently high concentrations of PAEs and OPEs suggest that packaging materials may represent a relevant contamination source, likely through migration processes.

**Fig. 3:**
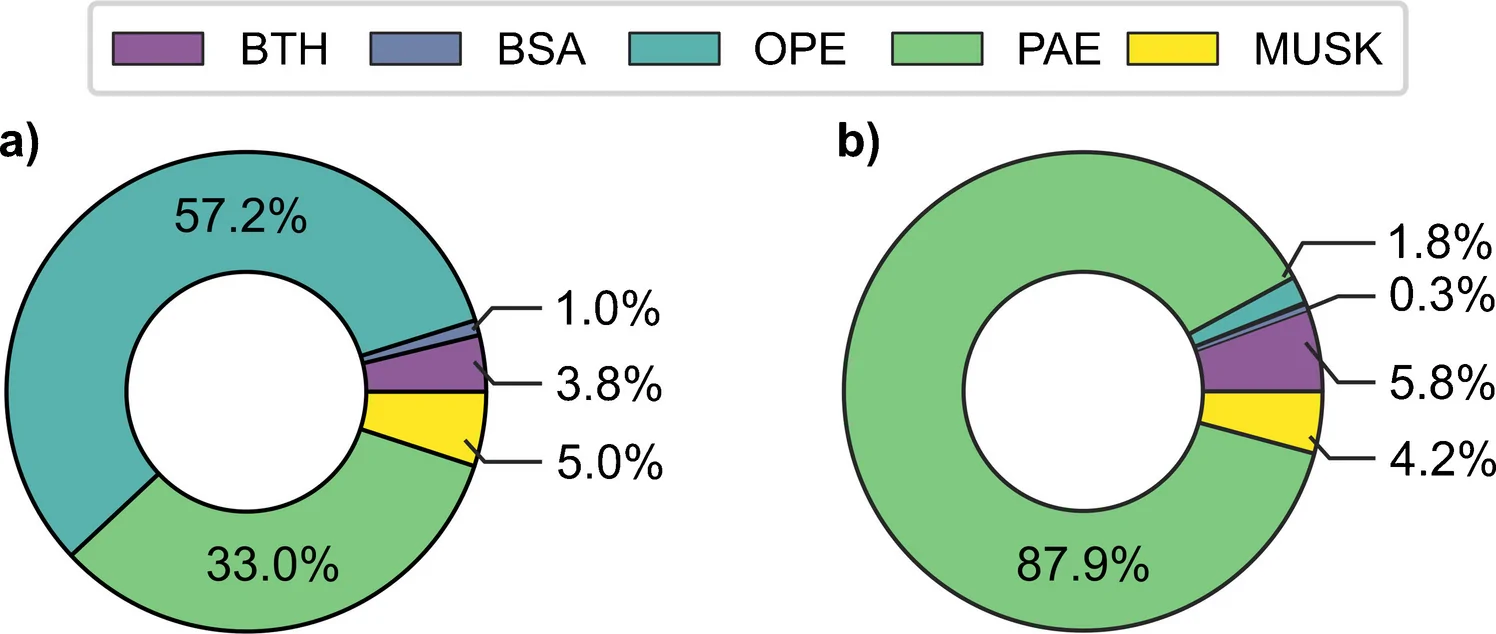
Total contribution of the HPVC families for **a** high-lipid species and **b** low-lipid species

To further explore species-related differences, chemical family contributions were compared between low-lipid (S11–S18) and high-lipid (S1–S10) species (Fig. 3). For low-lipid species, the contribution trend was ΣPAEs > > ΣBTHs > ΣMUSKs > ΣOPEs > ΣBSAs, whereas for high-lipid species, the trend was: ΣOPEs > ΣPAEs > > ΣMUSKs > ΣBTHs > ΣBSAs.

PAEs showed a particularly high relative contribution in meals based on low-lipid species (87.9%). Given their lipophilic nature and expected tendency to bioaccumulate in high-lipid tissues, this pattern suggests that external factors, such as processing conditions or packaging, may significantly influence their presence in these products. For high-lipid species, while OPEs appeared to be the predominant group (57.2%), this result was strongly influenced by the high OPE levels found in mussel-based meals. When mussel samples were excluded, PAEs also showed a substantial contribution for these species. The elevated PAEs concentrations in low-lipid species were largely driven by DnOP, suggesting a specific contamination source, potentially related to its use as a plasticizer in packaging materials.

### Human exposure and risk assessment

Dietary exposure to HPVCs through seafood consumption was estimated for six population subgroups: adult men (20–65 years old), adult women (20–65 years old), boys (10–19 years old), girls (10–19 years old), senior men (> 65 years old), and senior women (> 65 years old). Exposure was calculated under three scenarios—lower-bound (LB), middle-bound (MB), and upper-bound (UB)—to evaluate the potential risk associated with the consumption of each individual dish.

**Fig. 4:**
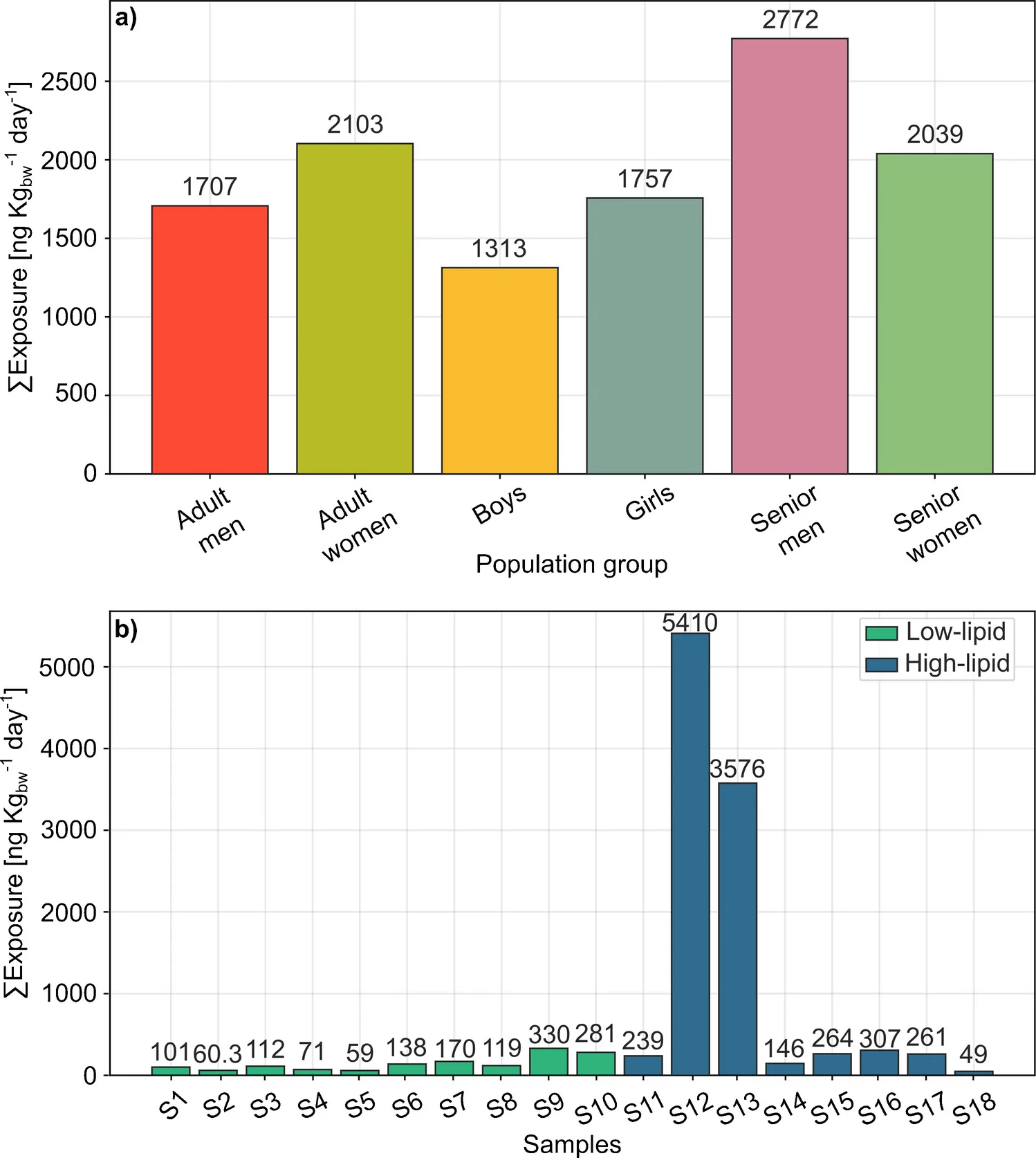
Exposure values in UB scenario. **a** Sum of the exposure per population group, considering all samples in ng kg_bw_^−1^ day^−1^. **b** Total exposure per sample, colored by low-lipid/high-lipid species, in ng kg_bw_^−1^ day^−1^

Figure 4 illustrates total dietary exposure (ng kg_bw_^−1^ day^−1^) under the UB scenario, presenting results by population subgroup (Fig. 4a) and by pre-cooked meal (Fig. 4b). Complete exposure data for all dishes, subgroups, and scenarios are provided in Supplementary Table S5.

Under the UB scenario, clear differences emerged among demographic groups. The highest exposure was estimated for senior men, followed by adult women, senior women, girls, adult men, and boys. When aggregated by gender, total exposure values were comparable between females and males (5899 vs males 5792 ng kg_bw_^−1^ day^−1^, respectively), indicating that variability was primarily age-related rather than gender-driven. This pattern aligns with seafood consumption data from the ENCAT 2023 survey [30], which identified senior men as the highest seafood-consuming subgroup. A comparable trend was reported by Castro et al. [26], who evaluated OPEs exposure from raw seafood and also identified senior men as the most exposed population group.

The highest exposure values were found for the hake-based meals S12 and S13 (Fig. 4), largely driven by the high contribution of PAEs. This effect was further enhanced by the high consumption frequency of hake across all population groups, as reported in the ENCAT 2003 [30]. When exposure was ranked by species-based meals, the overall order was hake > cod > mussels > sardines > salmon > shrimp > squid. This ranking differs from the consumption order reported in the ENCAT 2003 (hake > cod > squid > shrimp > sardine > salmon > mussels), indicating that dietary exposure is not solely determined by consumption volume. This discrepancy suggests that additional factors influence dietary exposure levels, including species lipid content (which may facilitate HPVC migration and accumulation), packaging materials, processing conditions, and seafood origin. All these factors ultimately affect analyte concentrations in the final meal.

Direct comparison with the literature is challenging due to the limited number of studies investigating pre-cooked seafood. Most available data refer to raw seafood samples and often focus on individual compounds or specific chemical families.

For example, Choi et al. [35] analyzed raw lake trout from the North American Great Lakes and reported Σ_12_OPEs exposure values between 6.5 and 31 ng kg_bw_^−1^ day^−1^. In the present study, under the UB scenario and the most exposed population subgroup (senior men), OPEs exposure values ranged from 0.08 to 72.8 ng kg_bw_^−1^ day^−1^. Although the upper bound slightly exceeds the values reported by Choi et al., the results remain the same order of magnitude.

Jia et al. [18] studied raw mollusks from the industrialized Bohai Sea (China) area and reported Σ_6_BTHs dietary exposure ranging from 76.1 to 124 ng kg_bw_^−1^ day^−1^. In our study, UB exposure values for BTHs in senior men ranged from 0.16 to 52.4 ng kg_bw_^−1^ day^−1^. The higher levels reported for the Bohai Sea are likely associated with the influence of industries near the sampling area, which may contribute to higher environmental contamination.

Similarly, Trabalón et al. [22] assessed BTH intake from raw seafood species in Tarragona (Spain) and reported a maximum exposure of 48 ng kg_bw_^−1^ day^−1^. The maximum value obtained in the present study (52.3 ng kg_bw_^−1^ day^−1^) is of the same order of magnitude.

Castro et al. [6] evaluated ΣOPEs, ΣBTHs, ΣBSAs, and ΣPAEs in raw fish from Tarragona (Spain), and reported UB exposure values between 8.0 and 68.9 ng kg_bw_^−1^ day^−1^. In contrast, total exposure in the present study ranged from 9.1 to 1294 ng kg_bw_^−1^ day^−1^ for senior men under the UB scenario. The higher values observed here were primarily driven by elevated PAE concentrations, suggesting that packaging contact and the cooking process may substantially contribute to overall exposure.

Only a few studies have evaluated total dietary intake across multiple food categories, including processed seafood. Poma et al. [39] assessed flame retardants and plasticizers in Belgian foodstuffs (including frozen, canned, smoked, and processed seafood) and reported total OPE intake of 103 ng kg_bw_^−1^ day^−1^. In the present study, total OPE exposure under the UB scenario reached 184 ng kg_bw_^−1^ day^−1^, which is comparable.

Similarly, a study conducted in Albany [40], including fish ball, cooked shrimp, frozen catfish, and smoked herring, estimated worst-case OPE intake of 135 ng kg_bw_^−1^ day^−1^, also comparable to the values obtained here.

Overall, despite differences in matrices, processing conditions, and geographical origin, the exposure values reported in this study fall within previously described ranges.

**Fig. 5:**
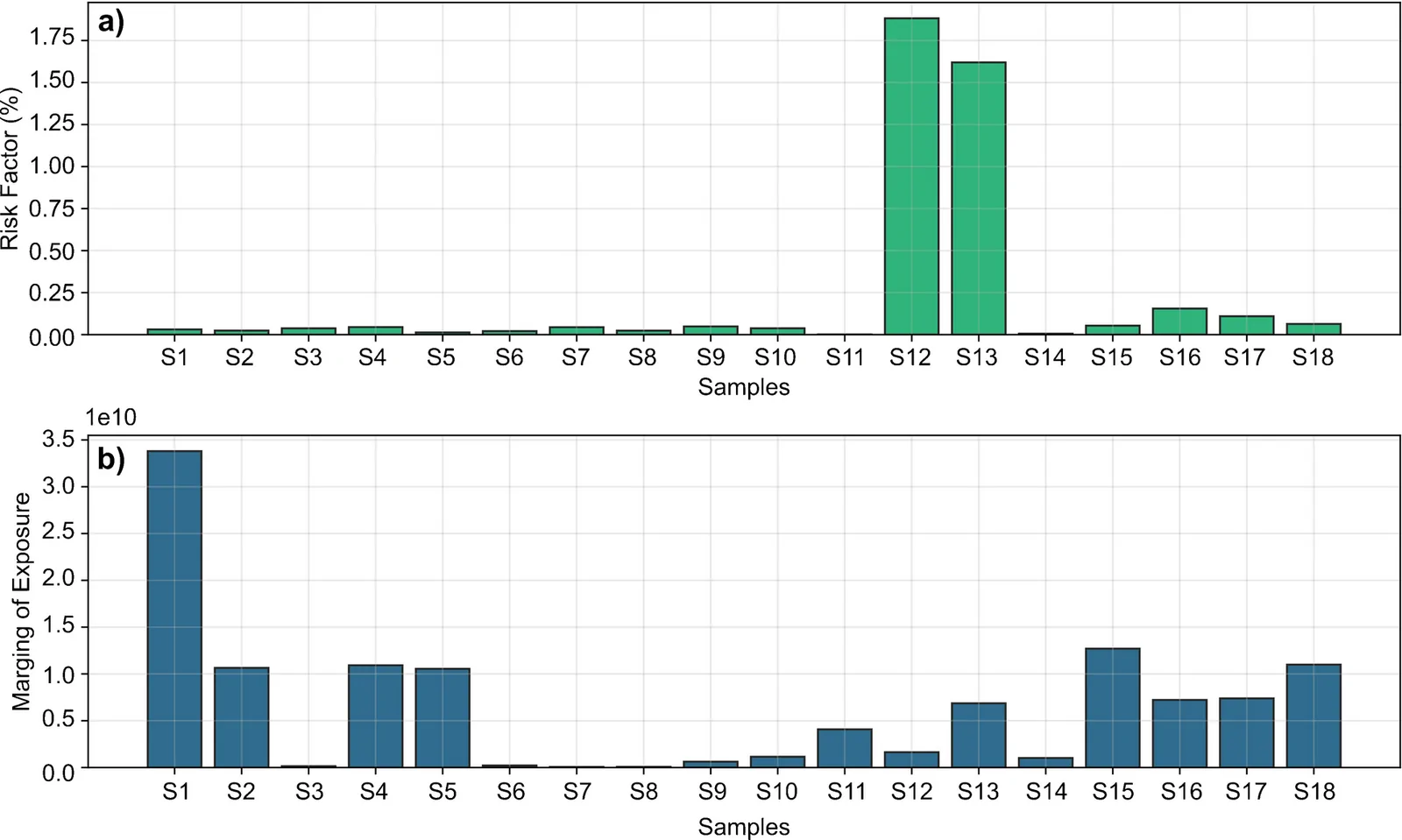
Risk assessment results for each sample for the most affected population group, senior men, considering only the UB scenario. Representation of the sum of compounds, separating target compounds according to carcinogenic risk in **a** margin of exposure and **b** risk factor

To assess potential health risks, non-genotoxic/non-carcinogenic and carcinogenic compounds were evaluated using the previously described equations. Figure 5 presents, as an example, the risk factor (hazard quotient) and margin of exposure (MOE) values for each pre-cooked meal in senior men under the UB scenario (worst-case conditions).

The non-genotoxic/non-carcinogenic compounds evaluated were TEP, BTH, TiBP, DMP, DEP, o-TSA, TCPP, p-TSA, DiBP, EHDPP, TPP, and DnOP (NOAEL values are provided in Supplementary Material S6). ADI values ranged from 2 × 10^3^ to 8 × 10^6^ ng kg_bw_^−1^ day^−1^. Individual compound exposure ranged from 0 to 1294 ng kg_bw_^−1^ day^−1^ (DnOP in a hake dish (S12), UB scenario, senior men). Hazard quotients were expressed as a percentage, with values below 100% indicating low risk. Overall, calculated risk factors were low: the geometric mean of the total compound sum per dish for senior men was 4 × 10^–3^%, well below the threshold of concern and consistent with previous studies [6]. The highest individual risk factor was observed for S12 (steamed hake with rice and shrimps in sauce), reaching 1.88% in senior men, still substantially below the threshold of concern. Full results for all dishes and subgroups are presented in Supplementary Material S7.

Compounds classified as carcinogenic included TBP, TCEP, TEHP, DEHA, and DEHP. Risk characterization was based on the benchmark dose (BMD) and expressed as MOE values (Table S6). According to EFSA criteria, MOE values higher than 10,000 indicate low concern. Under the UB scenario for senior men, MOE values ranged from 6.3 × 10^7^ to 3.4 × 10^10^, far exceeding the threshold of concern. Detailed MOE values are provided in Supplementary Table S8.

Considering both non-carcinogenic and carcinogenic compounds, the presence of the investigated HPVCs in the analyzed pre-cooked seafood meals does not represent a human health risk under the evaluated conditions. These findings are consistent with previous studies on raw seafood, which similarly reported no significant risk associated with consumption of these species [6, 35].

## Conclusions

In this study, five families of high production volume chemicals (HPVCs) were quantified in 18 commercially available pre-cooked seafood meals, providing for the first time, novel insight into contaminant levels in ready-to-eat seafood products. HPVCs were detected in most samples, following the overall concentration trend: ΣPAEs > ΣOPEs > ΣBTHs > ΣMUSKs > ΣBSAs. Phthalate esters (PAEs) were the predominant family, present in 72% of the analyzed meals.

Notably, concentrations of PAEs, OPEs, and BTHs in pre-cooked seafood were higher than those previously reported for raw fish from the same geographical area. These findings suggest that industrial processing and food packaging may represent significant contamination sources, likely through migration during cooking and storage. When evaluating the relationship between contaminant levels and lipid content, OPEs, MUSKs, and BSAs tended to show higher concentrations in high-lipid species, consistent with their lipophilic character and potential accumulation in fatty tissues. In contrast, PAEs were more abundant in low-lipid species, particularly hake. This pattern was largely driven by disproportionately high concentrations of DnOP in two hake-based meals (S12 and S13), highlighting the influence of specific contamination sources. Overall, contaminant levels appeared to be shaped by multiple interacting factors, including packaging materials, processing conditions, seafood origin, and species characteristics, although meals from the same species generally showed comparable distribution patterns.

Dietary exposure assessment indicated that consumption patterns were the primary determinant of exposure levels. Senior men were identified as the most exposed subgroup, reflecting their higher seafood intake, especially hake. Despite elevated concentrations in certain meals, particularly those with high PAE levels, risk characterization showed that both non-carcinogenic (hazard quotient) and carcinogenic (margin of exposure) assessments remained well within established safety thresholds. Although exposure values exceeded those reported for raw seafood in some cases—suggesting a potential contribution from packaging and processing—the overall presence of these HPVCs in pre-cooked seafood meals does not represent a significant health concern under the evaluated conditions.

## Supplementary Information

Below is the link to the electronic supplementary material.

Supplementary file1 (DOCX 206 KB)

## Data Availability

Data are available from the authors by request.

## References

[1] Domingo JL, Bocio A, Falcó G, Llobet JM. Benefits and risks of fish consumption. Toxicology. 2007;230:219–26. 10.1016/j.tox.2006.11.054. 17161894

[2] Kris-Etherton PM, Harris WS, Appel LJ. Fish consumption, fish oil, omega-3 fatty acids, and cardiovascular disease. Circulation. 2002;106:2747–57. 10.1161/01.CIR.0000038493.65177.94. 12438303

[3] Hooper L, Thompson RL, Harrison RA, Summerbell CD, Ness AR, Moore HJ, et al. Risks and benefits of omega 3 fats for mortality, cardiovascular disease, and cancer: systematic review. BMJ. 2006;332:752–60. 10.1136/bmj.38755.366331.2F. 16565093 PMC1420708

[4] Berger U, Glynn A, Holmström KE, Berglund M, Ankarberg EH, Törnkvist A. Fish consumption as a source of human exposure to perfluorinated alkyl substances in Sweden – analysis of edible fish from Lake Vättern and the Baltic Sea. Chemosphere. 2009;76:799–804. 10.1016/j.chemosphere.2009.04.044. 19457539

[5] Castro Ó, Borrull F, Pocurull E. High production volume chemicals in seafood: a review of analytical methods, occurrence and population risk. TrAC Trends Anal Chem. 2022;157:116743. 10.1016/j.trac.2022.116743.

[6] Castro Ó, Borrull S, Borrull F, Pocurull E. High production volume chemicals in the most consumed seafood species in Tarragona area (Spain): occurrence, exposure, and risk assessment. Food Chem Toxicol. 2023;173:113625. 10.1016/j.fct.2023.113625. 36682418

[7] Trabalón L, Cano-Sancho G, Pocurull E, Nadal M, Domingo JL, Borrull F. Exposure of the population of Catalonia (Spain) to musk fragrances through seafood consumption: risk assessment. Environ Res. 2015;143:116–22. 10.1016/j.envres.2015.04.007. 25913711

[8] Wang X, Wang W, Zhu Q, Wang Y, Liao C, Jiang G. Organophosphate esters in foodstuffs from multiple provinces in China: possible sources during food processing and implications for human exposure. J Agric Food Chem. 2022;70:8609–18. 10.1021/acs.jafc.2c03603. 35793444

[9] Ministerio de Agricultura, Pesca y Alimentación. Informe del consumo de alimentación en españa 2023. Madrid: Ministerio de Agricultura, Pesca y Alimentación; 2024. https://www.mapa.gob.es/dam/mapa/contenido/alimentacion/temas/consumo-y-tendencias-en-alimentacion/panel-de-consumo-alimentario/ultimos-datos/consumo-2023/informe_2023_alta.pdf. Accessed 3 Dec 2025

[10] Liao C, Kim U-J, Kannan K. A review of environmental occurrence, fate, exposure, and toxicity of benzothiazoles. Environ Sci Technol. 2018;52:5007–26. 10.1021/acs.est.7b05493. 29578695

[11] Ye T, Kang M, Huang Q, Fang C, Chen Y, Shen H, et al. Exposure to DEHP and MEHP from hatching to adulthood causes reproductive dysfunction and endocrine disruption in marine medaka (*Oryzias melastigma*). Aquat Toxicol. 2014;146:115–26. 10.1016/j.aquatox.2013.10.025. 24292025

[12] Fierens T, Servaes K, Van Holderbeke M, Geerts L, De Henauw S, Sioen I, et al. Analysis of phthalates in food products and packaging materials sold on the Belgian market. Food Chem Toxicol. 2012;50:2575–83. 10.1016/j.fct.2012.04.029. 22554646

[13] Freitas F, Cabrita MJ, Da Silva MG. A critical review of analytical methods for the quantification of phthalates esters in two important European food products: olive oil and wine. Molecules. 2023;28:7628. 10.3390/molecules28227628. 38005350 PMC10673500

[14] Castro V, Montes R, Quintana JB, Rodil R, Cela R. Determination of 18 organophosphorus flame retardants/plasticizers in mussel samples by matrix solid-phase dispersion combined to liquid chromatography-tandem mass spectrometry. Talanta. 2020;208:120470. 10.1016/j.talanta.2019.120470. 31816754

[15] Li J, Zhao L, Letcher RJ, Zhang Y, Jian K, Zhang J, et al. A review on organophosphate ester (OPE) flame retardants and plasticizers in foodstuffs: levels, distribution, human dietary exposure, and future directions. Environ Int. 2019;127:35–51. 10.1016/j.envint.2019.03.009. 30901640

[16] Castro Ó, Borrull S, Pocurull E, Borrull F. Determination of benzothiazoles, benzotriazoles and benzenesulfonamides in seafood using quick, easy, cheap, effective, rugged and safe extraction followed by gas chromatography - tandem mass spectrometry: method development and risk assessment. J Chromatogr A. 2023;1691:463841. 10.1016/j.chroma.2023.463841. 36739837

[17] Chen C-H, Chung W-H, Ding W-H. Determination of benzotriazole and benzothiazole derivatives in marketed fish by double-vortex-ultrasonic assisted matrix solid-phase dispersion and ultrahigh-performance liquid chromatography-high resolution mass spectrometry. Food Chem. 2020;333:127516. 10.1016/j.foodchem.2020.127516. 32683261

[18] Jia J, Zhu Q, Liu N, Liao C, Jiang G. Occurrence of and human exposure to benzothiazoles and benzotriazoles in mollusks in the Bohai Sea, China. Environ Int. 2019;130:104925. 10.1016/j.envint.2019.104925. 31247477

[19] Wang L, Asimakopoulos AG, Kannan K. Accumulation of 19 environmental phenolic and xenobiotic heterocyclic aromatic compounds in human adipose tissue. Environ Int. 2015;78:45–50. 10.1016/j.envint.2015.02.015. 25749637

[20] Picot Groz M, Martinez Bueno MJ, Rosain D, Fenet H, Casellas C, Pereira C, et al. Detection of emerging contaminants (UV filters, UV stabilizers and musks) in marine mussels from Portuguese coast by QuEChERS extraction and GC–MS/MS. Sci Total Environ. 2014;493:162–9. 10.1016/j.scitotenv.2014.05.062. 24946029

[21] Saraiva M, Cavalheiro J, Lanceleur L, Monperrus M. Synthetic musk in seafood products from south Europe using a quick, easy, cheap, effective, rugged and safe extraction method. Food Chem. 2016;200:330–5. 10.1016/j.foodchem.2016.01.017. 26830596

[22] Trabalón L, Nadal M, Borrull F, Pocurull E. Determination of benzothiazoles in seafood species by subcritical water extraction followed by solid-phase microextraction-gas chromatography-tandem mass spectrometry: estimating the dietary intake. Anal Bioanal Chem. 2017;409:5513–22. 10.1007/s00216-017-0487-3. 28687882

[23] Fierens T, Vanermen G, Van Holderbeke M, De Henauw S, Sioen I. Effect of cooking at home on the levels of eight phthalates in foods. Food Chem Toxicol. 2012;50:4428–35. 10.1016/j.fct.2012.09.004. 22985986

[24] Barbosa V, Maulvault AL, Alves RN, Kwadijk C, Kotterman M, Tediosi A, et al. Effects of steaming on contaminants of emerging concern levels in seafood. Food Chem Toxicol. 2018;118:490–504. 10.1016/j.fct.2018.05.047. 29787848

[25] Perelló G, Martí-Cid R, Castell V, Llobet JM, Domingo JL. Concentrations of polybrominated diphenyl ethers, hexachlorobenzene and polycyclic aromatic hydrocarbons in various foodstuffs before and after cooking. Food Chem Toxicol. 2009;47:709–15. 10.1016/j.fct.2008.12.030. 19162122

[26] Castro Ó, Pocurull E, Borrull F. Determination of organophosphate ester flame retardants and plasticisers in fish samples by QuEChERs followed by gas chromatography-tandem mass spectrometry. Exposure and risk assessment through fish consumption. J Chromatogr A. 2020;1626:461356. 10.1016/j.chroma.2020.461356. 32797836

[27] Ma Y, Cui K, Zeng F, Wen J, Liu H, Zhu F, et al. Microwave-assisted extraction combined with gel permeation chromatography and silica gel cleanup followed by gas chromatography–mass spectrometry for the determination of organophosphorus flame retardants and plasticizers in biological samples. Anal Chim Acta. 2013;786:47–53. 10.1016/j.aca.2013.04.062. 23790291

[28] Borrull S, Borrull F, Marcé RM, Pocurull E. Occurrence and intake risk assessment of high production volume chemicals in highly consumed seafood species. J Environ Sci (China). 2026;161:718–28. 10.1016/j.jes.2025.06.005. 41461518

[29] Xing Y, Gong X, Wang P, Wang Y, Wang L. Occurrence and release of organophosphite antioxidants and novel organophosphate esters from plastic food packaging. J Agric Food Chem. 2023;71:11599–606. 10.1021/acs.jafc.3c01138. 37470367

[30] ENCAT, Direcció General de Salut Pública. Avaluació de l’estat nutricional de la població catalana 2002/2003. Barcelona: Departament de Sanitat i Seguretat Social, Generalitat de Catalunya; 2004.

[31] Ministerio de Agricultura, Alimentación y Medio Ambiente. Guía nutricional del pescado y del marisco de las conservas. Madrid, España: Ministerio de Agricultura, Alimentación y Medio Ambiente; 2022 2025 Dec 3. https://burruezocongelados.es/public/assets/imagenes/guia-nutricional_pescados_mariscos_from.pdf. Accessed 3 Dec 2025

[32] Asante-Duah K. Public Health Risk Assessment for Human Exposure to Chemicals. 2nd ed. Springer Netherlands; 2017.

[33] European Food Safety Authority. Management of left‐censored data in dietary exposure assessment of chemical substances. EFS2. 2010;8. 10.2903/j.efsa.2010.1557

[34] Borrull S, Borrull F, Pocurull E, Marcé RM. Comparison of the presence of high production volume chemicals in farmed and wild fish highly consumed in Catalonia and their risk assessment. Chemosphere. 2024;365:143364. 10.1016/j.chemosphere.2024.143364. 39303794

[35] Choi Y, Hao C, Helm PA, Bhavsar SP, Kim SD. Organophosphate esters in Great Lakes fish: an improved analysis to assess concentrations and human exposure via consumption. Sci Total Environ. 2022;807:150981. 10.1016/j.scitotenv.2021.150981. 34666087

[36] Bi R, Meng W, Su G. Organophosphate esters (OPEs) in plastic food packaging: non-target recognition, and migration behavior assessment. Environ Int. 2023;177:108010. 10.1016/j.envint.2023.108010. 37307603

[37] Sundkvist AM, Olofsson U, Haglund P. Organophosphorus flame retardants and plasticizers in marine and fresh water biota and in human milk. J Environ Monit. 2010;12:943. 10.1039/b921910b. 20383376

[38] Alamri MS, Qasem AAA, Mohamed AA, Hussain S, Ibraheem MA, Shamlan G, et al. Food packaging’s materials: a food safety perspective. Saudi J Biol Sci. 2021;28:4490–9. 10.1016/j.sjbs.2021.04.047. 34354435 PMC8325021

[39] Poma G, Sales C, Bruyland B, Christia C, Goscinny S, Van Loco J, et al. Occurrence of organophosphorus flame retardants and plasticizers (PFRs) in Belgian foodstuffs and estimation of the dietary exposure of the adult population. Environ Sci Technol. 2018;52:2331–8. 10.1021/acs.est.7b06395. 29376341

[40] Wang Y, Kannan K. Concentrations and dietary exposure to organophosphate esters in foodstuffs from Albany, New York, United States. J Agric Food Chem. 2018;66:13525–32. 10.1021/acs.jafc.8b06114. 30525574

