# Supplementary material for: High production volume chemicals in pre-cooked seafood-based meals consumed by inhabitants of Tarragona (Catalonia, Spain): presence and risk assessment
Source: Anal Bioanal Chem. 2026 Apr 25;418(18):6045–58. doi: 10.1007/s00216-026-06521-2 (PMC13541955; doi:10.1007/s00216-026-06521-2)
Supplement: Supplementary file 1 [file 216_2026_6521_MOESM1_ESM.docx]

**SUPPLEMENTARY MATERIAL**

**High production volume chemicals in pre-cooked seafood-based meals consumed by inhabitants of Tarragona (Catalonia, Spain): presence and risk assessment**

Laura Borrell, Francesc Borrull, Carme Aguilar, Eva Pocurull

S1. GC-QqQ-MS/MS parameters.

S2. Mean seafood consumption (g day^-1^) obtained from the ENCAT 2003 survey.

S3. Instrumental and method quality parameters.

S4. Cumulative HPVC concentration in ng g^-1^ ww for each dish sample (S1-S18). Bar segments are colored according to each family's fraction.

S5. Total exposure values (ng Kg_bw_^-1^ day^-1^) of each pre-cooked dish for the different population’s subgroups, for the three different scenarios.

S6. NOAEL and BMD (ng Kg_bw_^-1^ day^-1^) values.

S7. Total risk factor (%, non-carcinogenic compounds) for each precooked dish across different population subgroups in three different scenarios.

S8. Total margin of exposure values for each precooked across different population subgroups in three different scenarios.

Table S1. GC-QqQ-MS/MS parameters.

| Analyte | Rt (min) | MRM Q | CE (eV) | MRM q | CE (eV) |
| --- | --- | --- | --- | --- | --- |
| TEP | 7.61 | 154.9 → 99.0 | 5 | 98.9 → 81.0 | 25 |
|  |  |  |  | 98.9 → 63.3 | 40 |
| d_4_-BTH | 9.21 | 139.0 → 70.0 | 51 | 139.0 → 84.0 | 35 |
|  |  |  |  | 139.0 → 112.0 | 21 |
| BTH | 9.24 | 135.1 → 108.0 | 21 | 135.1 → 82.0 | 33 |
|  |  |  |  | 135.1 → 69.0 | 49 |
| ClBT | 10.12 | 169.0 → 108.0 | 21 | 169.0 → 82.0 | 37 |
|  |  |  |  | 169.0 → 69.0 | 53 |
| TiBP | 10.61 | 99.0 → 81.0 | 25 | 98.9 → 63.0 | 45 |
|  |  |  |  | 98.9 → 47.0 | 50 |
| DPMI | 10.82 | 135.1 → 91.1 | 20 | 134.8 → 119.1 | 9 |
|  |  |  |  | 134.1 → 77.1 | 41 |
| DMP | 11.32 | 163.1 → 77.1 | 27 | 163.1 → 51.1 | 53 |
|  |  |  |  | 163.1 → 92.0 | 33 |
| d_27_-TBP | 11.69 | 103.1 → 83.1 | 25 | 102.9 → 63.0 | 45 |
|  |  |  |  | 146.9 → 62.1 | 20 |
| TBP | 11.86 | 98.9 → 80.9 | 25 | 98.9 → 63.0 | 25 |
|  |  |  |  | 98.9 → 47.1 | 45 |
| DEP | 12.24 | 149.0 → 65.1 | 25 | 149.0 → 93.1 | 15 |
|  |  |  |  | 149.0 → 121.0 | 15 |
| MeSBT | 12.71 | 180.8 → 148.0 | 15 | 179.8 → 135.0 | 19 |
|  |  |  |  | 181.0 → 108.0 | 41 |
| BSA | 13.01 | 157.1 → 93.1 | 5 | 157.1 → 77.0 | 25 |
|  |  |  |  | - | - |
| OHBT | 13.20 | 151.0 → 96.0 | 25 | 151.0 → 70.0 | 41 |
|  |  |  |  | 151.0 → 123.0 | 13 |
| ATII | 13.37 | 214.6 → 173.1 | 5 | 172.7 → 131.1 | 7 |
|  |  |  |  | 140.8 → 115.1 | 21 |
| o-TSA | 13.51 | 90.0 → 63.0 | 13 | 91.0 → 65.0 | 13 |
|  |  |  |  | 106.0 → 79.1 | 15 |
| HHCB | 13.52 | 243.0 → 213.2 | 7 | 257.0 → 213.2 | 5 |
|  |  |  |  | 272.1 → 257.0 | 5 |
| AHTN | 13.56 | 158.7 → 129.0 | 19 | 242.6 → 187.0 | 5 |
|  |  |  |  | 158.7 → 105.1 | 19 |
| TCPP | 13.61 | 125.0 → 99.0 | 20 | 124.0 → 81.0 | 25 |
|  |  |  |  | 98.9 → 81.0 | 10 |
| Me-p-TSA | 13.70 | 185.0 → 91.0 | 19 | 155.0 → 65.0 | 33 |
|  |  |  |  | 155.0 → 91.0 | 11 |
| Et-p-TSA | 13.808 | 184.0 → 91.0 | 29 | 155.0 → 91.0 | 11 |
|  |  |  |  | 91.0 → 65.0 | 15 |
| NH_2_BT | 13.72 | 150.0 → 96.0 | 27 | 150.0 → 70.0 | 43 |
|  |  |  |  | 150.0 → 123.0 | 15 |
| d_4_-p-TSA | 13.79 | 159.0 → 95.0 | 9 | 95.0 → 68.0 | 11 |
|  |  |  |  | 175.0 → 95.0 | 23 |
| p-TSA | 13.81 | 171.0 → 65.0 | 45 | 155.0 → 91.0 | 11 |
|  |  |  |  | 91.0 → 65.0 | 15 |
| DiBP | 13.83 | 149.1 → 65.1 | 27 | 149.1 → 93.1 | 19 |
|  |  |  |  | 149.1 → 121.0 | 15 |
| TCEP | 13.86 | 249.0 → 186.8 | 5 | 248.7 → 63.0 | 25 |
|  |  |  |  | 249.0 → 186.8 | 5 |
| D_15_-MX | 13.88 | 293.8 → 82.1 | 40 | 136.0 → 82.1 | 40 |
|  |  |  |  | 136.2 → 108.1 | 40 |
| DEHA | 15.97 | 129.1 → 55.1 | 17 | 129.1 → 101.1 | 5 |
|  |  |  |  | 129.1 → 111.1 | 5 |
| TEHP | 16.37 | 99.0 → 81.0 | 45 | 113.1 → 81.0 | 25 |
|  |  |  |  | - | - |
| d_4_-DEHP | 17.43 | 153.1 → 69.1 | 30 | 153.1 → 97.1 | 25 |
|  |  |  |  | 153.1 → 125.0 | 25 |
| DEHP | 17.44 | 149.1 → 65.1 | 29 | 149.1 → 93.1 | 15 |
|  |  |  |  | 149.1 → 121.0 | 15 |
| EHDPP | 17.46 | 251.2 → 77.1 | 20 | 251.2 → 152.2 | 20 |
|  |  |  |  | 251.2 → 175.1 | 20 |
| TPP | 18.09 | 325.8 → 170.0 | 15 | 325.8 → 232.9 | 15 |
|  |  |  |  | - | - |
| DnOP | 18.46 | 149.0 → 65.1 | 30 | 149.0 → 93.1 | 15 |
|  |  |  |  | 149.0 → 121.0 | 15 |
| TTP | 19.70 | 367.8 → 91.0 | 35 | 368.8 → 164.8 | 30 |
|  |  |  |  | 367.8 → 243.2 | 30 |

CE, collision energy

Rt, retention time

Q, quantification transition

q, qualification transition

Table S2. Mean seafood consumption (g day^-1^) obtained from the ENCAT 2003 survey.

|  | Boys  10-19 | Adult men  20-65 | Senior men  >65 | Girls  10-19 | Adult women  20-65 | Senior women  >65 |
| --- | --- | --- | --- | --- | --- | --- |
| Hake | 7.82 | 15.03 | 23.02 | 10.84 | 14.49 | 14.56 |
| Sardine | 0.99 | 2.92 | 2.60 | 2.08 | 2.69 | 4.70 |
| Tuna | 0.71 | 1.62 | 1.07 | 0.00 | 1.45 | 0.52 |
| Sole | 6.22 | 4.84 | 3.65 | 2.44 | 5.28 | 5.17 |
| Cod | 2.13 | 4.18 | 8.08 | 0.60 | 4.61 | 8.15 |
| Mackerel | 0.36 | 1.13 | 0.50 | 0.32 | 1.27 | 2.86 |
| Salmon | 3.30 | 1.80 | 2.23 | 1.00 | 3.00 | 1.14 |
| Mussel | 1.26 | 0.97 | 2.06 | 0.00 | 1.84 | 0.67 |
| Squid | 1.88 | 3.17 | 3.18 | 5.18 | 3.17 | 0.77 |
| Shrimp | 2.71 | 2.83 | 2.42 | 2.94 | 3.44 | 1.68 |

Table S3. Instrumental and method quality parameters.

|  | Instrumental parameters | | | Method parameters | | | |
| --- | --- | --- | --- | --- | --- | --- | --- |
| Compound | linear range (µg L^-1^) | Correlation coefficient, r^2^ | LOD (µg L^-1^) | Rapp% | ME% | MDL (ng g^-1^ ww) | MQL (ng g^-1^ ww) |
| BTH | 5 – 250 | 0.9985 | 0.01 | 39 – 94 | -92 – 80 | 0.038 – 0.215 | 19.15 – 107.5 |
| ClBT | 0.05 – 250 | 0.9977 | 0.005 | 41 – 68 | -66 – 9 | 0.026 – 0.117 | 0.265 – 1.173 |
| MeSBT | 0.1 – 250 | 0.9959 | 0.01 | 34 – 64 | -77 – -12 | 0.056 – 0.272 | 0.563 – 2.716 |
| OHBT | 0.5 – 250 | 0.9989 | 0.25 | 35 – 85 | -59 – 34 | 1.059 – 10.25 | 2.118 – 20.51 |
| NH_2_BT | 5 – 100 | 0.9977 | 0.25 | 42 – 113 | -81 – 57 | 1.000 – 5.060 | 20.00 – 101.2 |
| BSA | 0.5 – 250 | 0.9980 | 0.1 | 50 – 98 | -42 – 87 | 0.375 – 1.214 | 1.837 – 6.072 |
| o-TSA | 1 – 250 | 0.9968 | 0.25 | 44 – 92 | -56 – 61 | 1.047 – 15.88 | 4.189 – 63.52 |
| Me-p-TSA | 0.1 – 250 | 0.9940 | 0.007 | 41 – 101 | -80 – 58 | 0.043 – 0.092 | 0.609 – 1.320 |
| Et-p-TSA | 0.05 – 100 | 0.9977 | 0.005 | 41 – 105 | -57 – 79 | 0.018 – 0.064 | 0.184 – 0.637 |
| p-TSA | 1 – 250 | 0.9997 | 0.025 | 51 – 85 | -64 – 28 | 0.100 – 0.461 | 4.000 – 18.43 |
| TEP | 0.1 – 250 | 0.9999 | 0.005 | 43 – 135 | -25 – 74 | 0.021 – 0.101 | 0.414 – 1.032 |
| TBP | 0.005 – 100 | 0.9987 | 0.001 | 47 – 80 | -30 – 6 | 0.006 – 0.017 | 0.030 – 0.086 |
| TiBP | 0.005 – 100 | 0.9997 | 0.001 | 45 – 119 | -38 – 49 | 0.005 – 0.011 | 0.024 – 0.056 |
| TCPP | 0.5 – 250 | 0.9997 | 0.01 | 49 – 76 | -64 – -4 | 0.047 – 0.157 | 2.338 – 7.828 |
| TCEP | 1 – 250 | 0.9991 | 0.01 | 50 – 85 | -81 – 14 | 0.041 – 0.153 | 4.138 – 22.44 |
| TEHP | 0.005 – 100 | 0.9990 | 0.001 | 39 – 98 | -39 – 28 | 0.005 – 0.026 | 0.025 – 0.132 |
| EHDPP | 0.005 – 250 | 0.9997 | 0.001 | 38 – 56 | -72 – 1 | 0.008 – 0.026 | 0.035 – 0.128 |
| TPP | 0.005 – 250 | 0.9999 | 0.001 | 37 – 67 | -47 – -9 | 0.007 – 0.028 | 0.037 – 0.139 |
| TTP | 0.1 – 250 | 0.9991 | 0.1 | 49 – 116 | -55 – 61 | 0.310 – 1.811 | 0.310 – 2.131 |
| DMP | 0.005 – 250 | 0.9986 | 0.01 | 39 – 95 | -44 – 62 | 0.038 – 0.113 | 0.019 – 0.057 |
| DEP | 0.005 – 100 | 0.9993 | 0.001 | 42 – 129 | -41 – 66 | 0.004 – 0.012 | 0.021 – 0.062 |
| DiBP | 0.005 – 100 | 0.9932 | 0.01 | 41 – 125 | -66 – 61 | 0.048 – 0.148 | 0.024 – 0.074 |
| DEHA | 0.05 – 250 | 0.9984 | 0.01 | 44 – 131 | -42 – 85 | 0.041 – 0.116 | 0.207 – 0.581 |
| DEHP | 0.005 – 100 | 0.9983 | 0.007 | 42 – 110 | -61 – 87 | 0.032 – 0.128 | 0.023 – 0.091 |
| DnOP | 1 – 100 | 0.9982 | 0.025 | 48 – 134 | -65 – 77 | 0.078 – 0.400 | 3.130 – 8.302 |
| DPMI | 1 – 250 | 0.9996 | 0.007 | 41 – 140 | 1 – 85 | 0.021 – 0.086 | 3.051 – 12.23 |
| ATII | 0.5 – 250 | 0.9999 | 0.01 | 43 – 99 | -25 – 45 | 0.036 – 0.169 | 1.818 – 8.445 |
| HHCB | 0.01 – 250 | 0.9999 | 0.001 | 28 – 96 | -49 – 36 | 0.038 – 0.188 | 0.038 – 0.207 |
| AHTN | 1 – 250 | 0.9998 | 0.05 | 39 – 84 | -51 – 20 | 0.344 – 1.518 | 4.286 – 30.36 |

LOD, limit of detection

Rapp, apparent recovery

ME, matrix effect

MDL, method detection limit

MQL, method quantification limit.

Figure S4. Cumulative HPVC concentration in ng g^-1^ ww for each dish sample (S1-S18). Bar segments are colored according to each family's fraction.

.

Tables S5. Total exposure values (ng Kg_bw_^-1^ day^-1^) of each pre-cooked dish for the different population’s subgroups, for the three different scenarios.

| **Population group** |  | **S1** | **S2** | **S3** | **S4** | **S5** | **S6** | **S7** | **S8** | **S9** | **S10** | **S11** | **S12** | **S13** | **S14** | **S15** | **S16** | **S17** | **S18** |
| --- | --- | --- | --- | --- | --- | --- | --- | --- | --- | --- | --- | --- | --- | --- | --- | --- | --- | --- | --- |
| **BOYS (10-19)** | LB^a^ | 10.5 | 5.4 | 19.8 | 12.7 | 16.7 | 36.5 | 11.4 | 7.9 | 71.4 | 59.7 | 19.5 | 560.6 | 369.0 | 12.9 | 23.5 | 27.5 | 23.1 | 4.1 |
|  | MB^b^ | 11.0 | 6.2 | 20.1 | 12.8 | 16.9 | 38.6 | 11.7 | 8.1 | 71.4 | 60.3 | 22.0 | 560.8 | 370.0 | 13.1 | 23.7 | 27.7 | 23.4 | 4.2 |
|  | UB^c^ | 11.6 | 6.9 | 20.6 | 13.1 | 17.2 | 40.7 | 11.9 | 8.3 | 71.4 | 60.8 | 24.8 | 561.4 | 371.1 | 13.2 | 24.0 | 27.9 | 23.7 | 4.4 |
| **GIRLS**  **(10-19)** | LB | 33.9 | 17.5 | 25.2 | 16.1 | 5.9 | 12.9 | 28.1 | 19.3 | 0.0 | 0.0 | 31.6 | 909.5 | 598.6 | 4.3 | 7.8 | 9.1 | 7.6 | 1.3 |
|  | MB | 35.5 | 19.9 | 25.6 | 16.3 | 6.0 | 13.7 | 28.7 | 19.9 | 0.0 | 0.0 | 35.7 | 909.8 | 600.3 | 4.3 | 7.8 | 9.1 | 7.7 | 1.4 |
|  | UB | 37.4 | 22.4 | 26.2 | 16.6 | 6.1 | 14.4 | 29.3 | 20.5 | 0.0 | 0.0 | 40.2 | 910.7 | 602.1 | 4.4 | 7.9 | 9.2 | 7.8 | 1.5 |
| **ADULT MEN (20-65)** | LB | 13.3 | 6.8 | 15.5 | 9.9 | 6.8 | 14.9 | 25.2 | 17.3 | 41.1 | 34.4 | 28.0 | 806.0 | 530.4 | 19.0 | 34.5 | 40.3 | 33.9 | 6.0 |
|  | MB | 13.9 | 7.8 | 15.7 | 10.0 | 6.9 | 15.7 | 25.7 | 17.8 | 41.1 | 34.7 | 31.6 | 806.2 | 532.0 | 19.2 | 34.8 | 40.6 | 34.4 | 6.2 |
|  | UB | 14.6 | 8.8 | 16.1 | 10.2 | 7.0 | 16.6 | 26.3 | 18.4 | 41.1 | 35.0 | 35.6 | 807.1 | 533.5 | 19.4 | 35.2 | 40.9 | 34.7 | 6.5 |
| **ADULT**  **WOMEN**  **(20-65)** | LB | 15.9 | 8.2 | 22.5 | 14.4 | 13.6 | 29.7 | 27.8 | 19.1 | 93.3 | 78.0 | 32.3 | 929.8 | 611.9 | 25.1 | 45.6 | 53.2 | 44.7 | 7.9 |
|  | MB | 16.6 | 9.3 | 22.9 | 14.6 | 13.8 | 31.4 | 28.3 | 19.7 | 93.3 | 78.8 | 36.5 | 930.0 | 613.7 | 25.3 | 46.0 | 53.6 | 45.4 | 8.2 |
|  | UB | 17.5 | 10.5 | 23.4 | 14.9 | 14.0 | 33.1 | 29.0 | 20.2 | 93.3 | 79.5 | 41.1 | 931.0 | 615.5 | 25.7 | 46.4 | 54.0 | 45.8 | 8.5 |
| **SENIOR MEN (>65)** | LB | 13.9 | 7.2 | 13.9 | 8.9 | 8.8 | 19.3 | 23.5 | 16.2 | 91.3 | 76.4 | 44.9 | 1292.0 | 850.3 | 38.4 | 69.9 | 81.6 | 68.5 | 12.1 |
|  | MB | 14.6 | 8.2 | 14.1 | 9.0 | 9.0 | 20.4 | 24.0 | 16.6 | 91.4 | 77.1 | 50.7 | 1292.3 | 852.7 | 38.9 | 70.5 | 82.1 | 69.6 | 12.5 |
|  | UB | 15.4 | 9.2 | 14.4 | 9.1 | 9.1 | 21.4 | 24.5 | 17.1 | 91.4 | 77.8 | 57.1 | 1293.7 | 855.3 | 39.3 | 71.2 | 82.8 | 70.2 | 13.1 |
| **SENIOR**  **WOMEN (>65)** | LB | 3.7 | 1.9 | 10.6 | 6.8 | 5.0 | 10.9 | 46.9 | 32.3 | 32.9 | 27.5 | 31.5 | 904.5 | 595.2 | 42.9 | 78.0 | 91.1 | 76.5 | 13.5 |
|  | MB | 3.9 | 2.2 | 10.8 | 6.9 | 5.1 | 11.5 | 47.9 | 33.3 | 32.9 | 27.8 | 35.5 | 904.7 | 596.9 | 43.4 | 78.7 | 91.7 | 77.7 | 14.0 |
|  | UB | 4.1 | 2.5 | 11.1 | 7.0 | 5.2 | 12.2 | 49.0 | 34.2 | 32.9 | 28.0 | 39.9 | 905.7 | 598.7 | 43.9 | 79.5 | 92.4 | 78.4 | 14.6 |

^a^ Lower-bound scenario

^b^ Middle-bound scenario

^c^ Upper-bound scenario

Table S6. NOAEL and BMD (ng Kg_bw_^-1^ day^-1^) values.

| Compound | NOAEL [ng/Kg b.w day] | BMD [ng/Kg b.w day] |
| --- | --- | --- |
| TEP | 125000000 | - |
| BTH | 5100000 | - |
| ClBT | - | - |
| TiBP | 684000 | - |
| DPMI | - | - |
| DMP | 7500000 | - |
| TBP | - | 9000000 |
| DEP | 800000000 | - |
| MeSBT | - | - |
| BSA | - | - |
| OHBT | - | - |
| ATII | - | - |
| o-TSA | 200000 | - |
| HHCB | - | - |
| AHTN | - | - |
| TCPP | 1000000 | - |
| Me-p-TSA | - | - |
| Et-p-TSA | - | - |
| NH_2_BT | - | - |
| p-TSA | 2480000 | - |
| DiBP | 1770000 | - |
| TCEP | - | 20000000 |
| DEHA | - | 1200000 |
| TEHP | - | 3200000 |
| DEHP | - | 14000000 |
| EHDPP | 208000 | - |
| TPP | 1170000 | - |
| DnOP | 7000000 | - |
| TTP | 10000000 | - |

Tables S7. Total risk factor (%, non-carcinogenic compounds) for each precooked dish across different population subgroups in three different scenarios.

| **Population group** |  | **S1** | **S2** | **S3** | **S4** | **S5** | **S6** | **S7** | **S8** | **S9** | **S10** | **S11** | **S12** | **S13** | **S14** | **S15** | **S16** | **S17** | **S18** |
| --- | --- | --- | --- | --- | --- | --- | --- | --- | --- | --- | --- | --- | --- | --- | --- | --- | --- | --- | --- |
| **BOYS**  **(10-19)** | LB^a^ | 0.023 | 0.017 | 0.046 | 0.063 | 0.025 | 0.031 | 0.021 | 0.010 | 0.038 | 0.027 | 0.000 | 0.809 | 0.699 | 0.000 | 0.016 | 0.050 | 0.029 | 0.013 |
|  | MB^b^ | 0.023 | 0.017 | 0.046 | 0.064 | 0.025 | 0.035 | 0.021 | 0.011 | 0.038 | 0.028 | 0.000 | 0.809 | 0.701 | 0.001 | 0.017 | 0.051 | 0.032 | 0.016 |
|  | UB^c^ | 0.023 | 0.018 | 0.054 | 0.064 | 0.025 | 0.039 | 0.022 | 0.012 | 0.038 | 0.029 | 0.001 | 0.817 | 0.703 | 0.002 | 0.018 | 0.052 | 0.037 | 0.021 |
| **GIRLS**  **(10-19)** | LB | 0.075 | 0.053 | 0.058 | 0.080 | 0.009 | 0.011 | 0.053 | 0.025 | 0.000 | 0.000 | 0.000 | 1.312 | 1.134 | 0.000 | 0.005 | 0.016 | 0.010 | 0.004 |
|  | MB | 0.076 | 0.056 | 0.058 | 0.081 | 0.009 | 0.013 | 0.053 | 0.027 | 0.000 | 0.000 | 0.001 | 1.312 | 1.137 | 0.000 | 0.006 | 0.017 | 0.010 | 0.005 |
|  | UB | 0.076 | 0.059 | 0.068 | 0.082 | 0.009 | 0.014 | 0.053 | 0.029 | 0.000 | 0.000 | 0.001 | 1.325 | 1.140 | 0.001 | 0.006 | 0.017 | 0.012 | 0.007 |
| **ADULT MEN (20-65)** | LB | 0.030 | 0.021 | 0.036 | 0.049 | 0.010 | 0.013 | 0.047 | 0.023 | 0.022 | 0.016 | 0.000 | 1.162 | 1.005 | 0.000 | 0.023 | 0.073 | 0.042 | 0.019 |
|  | MB | 0.030 | 0.022 | 0.036 | 0.050 | 0.010 | 0.014 | 0.047 | 0.024 | 0.022 | 0.016 | 0.000 | 1.162 | 1.008 | 0.001 | 0.025 | 0.075 | 0.046 | 0.023 |
|  | UB | 0.030 | 0.023 | 0.042 | 0.050 | 0.010 | 0.016 | 0.047 | 0.026 | 0.022 | 0.017 | 0.001 | 1.174 | 1.011 | 0.003 | 0.026 | 0.077 | 0.054 | 0.031 |
| **ADULT**  **WOMEN**  **(20-65)** | LB | 0.035 | 0.025 | 0.052 | 0.071 | 0.020 | 0.026 | 0.052 | 0.025 | 0.050 | 0.036 | 0.000 | 1.341 | 1.159 | 0.000 | 0.031 | 0.096 | 0.056 | 0.026 |
|  | MB | 0.035 | 0.026 | 0.052 | 0.072 | 0.020 | 0.029 | 0.052 | 0.027 | 0.050 | 0.037 | 0.001 | 1.341 | 1.162 | 0.002 | 0.033 | 0.099 | 0.061 | 0.031 |
|  | UB | 0.035 | 0.028 | 0.061 | 0.073 | 0.020 | 0.032 | 0.052 | 0.028 | 0.050 | 0.039 | 0.001 | 1.355 | 1.166 | 0.004 | 0.035 | 0.101 | 0.071 | 0.041 |
| **SENIOR MEN (>65)** | LB | 0.031 | 0.022 | 0.032 | 0.044 | 0.013 | 0.017 | 0.044 | 0.021 | 0.049 | 0.035 | 0.000 | 1.863 | 1.611 | 0.000 | 0.047 | 0.147 | 0.086 | 0.039 |
|  | MB | 0.031 | 0.023 | 0.032 | 0.044 | 0.013 | 0.019 | 0.044 | 0.023 | 0.049 | 0.036 | 0.001 | 1.863 | 1.615 | 0.003 | 0.050 | 0.151 | 0.094 | 0.047 |
|  | UB | 0.031 | 0.024 | 0.038 | 0.045 | 0.013 | 0.021 | 0.044 | 0.024 | 0.049 | 0.038 | 0.001 | 1.882 | 1.620 | 0.006 | 0.054 | 0.155 | 0.110 | 0.064 |
| **SENIOR**  **WOMEN (>65)** | LB | 0.008 | 0.006 | 0.024 | 0.034 | 0.007 | 0.009 | 0.088 | 0.042 | 0.018 | 0.013 | 0.000 | 1.304 | 1.128 | 0.000 | 0.053 | 0.164 | 0.096 | 0.044 |
|  | MB | 0.008 | 0.006 | 0.024 | 0.034 | 0.007 | 0.011 | 0.088 | 0.045 | 0.018 | 0.013 | 0.001 | 1.304 | 1.131 | 0.003 | 0.056 | 0.169 | 0.105 | 0.053 |
|  | UB | 0.008 | 0.006 | 0.029 | 0.035 | 0.007 | 0.012 | 0.088 | 0.048 | 0.018 | 0.014 | 0.001 | 1.318 | 1.134 | 0.007 | 0.060 | 0.174 | 0.122 | 0.071 |

^a^ Lower-bound scenario

^b^ Middle-bound scenario

^c^ Upper-bound scenario

Tables S8. Total margin of exposure values for each precooked across different population subgroups in three different scenarios.

| **Population group** |  | **S1** | **S2** | **S3** | **S4** | **S5** | **S6** | **S7** | **S8** | **S9** | **S10** | **S11** | **S12** | **S13** | **S14** | **S15** | **S16** | **S17** | **S18** |
| --- | --- | --- | --- | --- | --- | --- | --- | --- | --- | --- | --- | --- | --- | --- | --- | --- | --- | --- | --- |
| **BOYS**  **(10-19)** | LB^a^ | 6E+09 | 3E+10 | 5E+09 | 1E+08 | 3E+08 | 1E+08 | 5E+09 | 8E+09 | 8E+08 | 1E+09 | 9E+09 | 7E+09 | 0E+00 | 6E+10 | 9E+06 | 4E+10 | 5E+10 | 4E+10 |
|  | MB^b^ | 9E+10 | 3E+10 | 1E+08 | 2E+10 | 1E+10 | 1E+08 | 2E+08 | 2E+08 | 8E+08 | 1E+09 | 2E+10 | 8E+09 | 3E+10 | 2E+10 | 8E+10 | 4E+10 | 3E+10 | 7E+10 |
|  | UB^c^ | 4E+10 | 1E+10 | 1E+08 | 8E+09 | 6E+09 | 1E+08 | 1E+08 | 2E+08 | 8E+08 | 1E+09 | 9E+09 | 4E+09 | 2E+10 | 3E+09 | 4E+10 | 2E+10 | 2E+10 | 3E+10 |
| **GIRLS**  **(10-19)** | LB | 2E+09 | 8E+09 | 4E+09 | 1E+08 | 9E+08 | 3E+08 | 2E+09 | 3E+09 | 0E+00 | 0E+00 | 6E+09 | 4E+09 | 0E+00 | 2E+11 | 3E+07 | 1E+11 | 1E+11 | 1E+11 |
|  | MB | 3E+10 | 9E+09 | 1E+08 | 1E+10 | 3E+10 | 3E+08 | 7E+07 | 9E+07 | 0E+00 | 0E+00 | 1E+10 | 5E+09 | 2E+10 | 7E+10 | 2E+11 | 1E+11 | 9E+10 | 2E+11 |
|  | UB | 3E+10 | 3E+10 | 3E+10 | 3E+10 | 3E+10 | 3E+10 | 3E+10 | 3E+10 | 3E+10 | 3E+10 | 3E+10 | 3E+10 | 3E+10 | 3E+10 | 3E+10 | 3E+10 | 3E+10 | 3E+10 |
| **ADULT MEN (20-65)** | LB | 4E+09 | 2E+10 | 6E+09 | 2E+08 | 8E+08 | 3E+08 | 2E+09 | 4E+09 | 1E+09 | 3E+09 | 7E+09 | 5E+09 | 0E+00 | 4E+10 | 6E+06 | 2E+10 | 3E+10 | 3E+10 |
|  | MB | 7E+10 | 2E+10 | 2E+08 | 2E+10 | 3E+10 | 3E+08 | 8E+07 | 1E+08 | 1E+09 | 3E+09 | 1E+10 | 5E+09 | 2E+10 | 2E+10 | 5E+10 | 3E+10 | 2E+10 | 4E+10 |
|  | UB | 4E+10 | 1E+10 | 1E+08 | 1E+10 | 1E+10 | 3E+08 | 6E+07 | 7E+07 | 1E+09 | 3E+09 | 7E+09 | 3E+09 | 1E+10 | 2E+09 | 3E+10 | 1E+10 | 1E+10 | 2E+10 |
| **ADULT**  **WOMEN**  **(20-65)** | LB | 4E+09 | 2E+10 | 4E+09 | 1E+08 | 4E+08 | 1E+08 | 2E+09 | 3E+09 | 6E+08 | 1E+09 | 6E+09 | 4E+09 | 0E+00 | 3E+10 | 5E+06 | 2E+10 | 3E+10 | 2E+10 |
|  | MB | 6E+10 | 2E+10 | 1E+08 | 1E+10 | 1E+10 | 1E+08 | 8E+07 | 9E+07 | 6E+08 | 1E+09 | 1E+10 | 5E+09 | 2E+10 | 1E+10 | 4E+10 | 2E+10 | 1E+10 | 3E+10 |
|  | UB | 3E+10 | 3E+10 | 3E+10 | 3E+10 | 3E+10 | 3E+10 | 3E+10 | 3E+10 | 3E+10 | 3E+10 | 3E+10 | 3E+10 | 3E+10 | 3E+10 | 3E+10 | 3E+10 | 3E+10 | 3E+10 |
| **SENIOR MEN (>65)** | LB | 4E+09 | 2E+10 | 7E+09 | 2E+08 | 6E+08 | 2E+08 | 3E+09 | 4E+09 | 6E+08 | 1E+09 | 4E+09 | 3E+09 | 0E+00 | 2E+10 | 3E+06 | 1E+10 | 2E+10 | 1E+10 |
|  | MB | 7E+10 | 2E+10 | 2E+08 | 2E+10 | 2E+10 | 2E+08 | 9E+07 | 1E+08 | 6E+08 | 1E+09 | 8E+09 | 3E+09 | 1E+10 | 8E+09 | 3E+10 | 1E+10 | 9E+09 | 2E+10 |
|  | UB | 3E+10 | 1E+10 | 1E+08 | 1E+10 | 1E+10 | 2E+08 | 6E+07 | 7E+07 | 6E+08 | 1E+09 | 4E+09 | 2E+09 | 7E+09 | 1E+09 | 1E+10 | 7E+09 | 7E+09 | 1E+10 |
| **SENIOR**  **WOMEN (>65)** | LB | 2E+10 | 7E+10 | 9E+09 | 3E+08 | 1E+09 | 4E+08 | 1E+09 | 2E+09 | 2E+09 | 3E+09 | 6E+09 | 4E+09 | 0E+00 | 2E+10 | 3E+06 | 1E+10 | 1E+10 | 1E+10 |
|  | MB | 3E+11 | 8E+10 | 3E+08 | 3E+10 | 4E+10 | 4E+08 | 4E+07 | 6E+07 | 2E+09 | 3E+09 | 1E+10 | 5E+09 | 2E+10 | 7E+09 | 2E+10 | 1E+10 | 8E+09 | 2E+10 |
|  | UB | 3E+10 | 3E+10 | 3E+10 | 3E+10 | 3E+10 | 3E+10 | 3E+10 | 3E+10 | 3E+10 | 3E+10 | 3E+10 | 3E+10 | 3E+10 | 3E+10 | 3E+10 | 3E+10 | 3E+10 | 3E+10 |

^a^ Lower-bound scenario

^b^ Middle-bound scenario

^c^ Upper-bound scenario
